# Carolacton Treatment Causes Delocalization of the Cell Division Proteins PknB and DivIVa in *Streptococcus mutans in vivo*

**DOI:** 10.3389/fmicb.2016.00684

**Published:** 2016-05-11

**Authors:** Michael Reck, Irene Wagner-Döbler

**Affiliations:** Department of Microbiology, Microbial Communication, Helmholtz Centre for Infection ResearchBraunschweig, Germany

**Keywords:** protein localization, fluorescent fusion protein, inducible expression vectors, streptococci, single cell analysis, Carolacton, cell division, serine/threonine protein kinase

## Abstract

The small inhibitory molecule Carolacton has been shown to cause chain formation and bulging in Streptococci, suggesting a defect in cell division, but it is not known how cell division is impaired on a molecular level. Fluorescent fusion proteins have successfully been applied to visualize protein localization and dynamics *in vivo* and have revolutionized our understanding of cell wall growth, cell division, chromosome replication and segregation. However, in Streptococci the required vectors are largely lacking. We constructed vectors for chromosomal integration and inducible expression of fluorescent fusion proteins based on GFP+ in *S. mutans*. Their applicability was verified using four proteins with known localization in the cell. We then determined the effect of Carolacton on the subcellular localization of GFP+ fusions of the cell division protein DivIVa and the serine-threonine protein kinase PknB. Carolacton caused a significant delocalization of these proteins from midcell, in accordance with a previous study demonstrating the Carolacton insensitive phenotype of a *pknB* deletion strain. Carolacton treated cells displayed an elongated phenotype, increased septum formation and a severe defect in daughter cell separation. GFP+ fusions of two hypothetical proteins (SMU_503 and SMU_609), that had previously been shown to be the most strongly upregulated genes after Carolacton treatment, were found to be localized at the septum in midcell, indicating their role in cell division. These findings highlight the importance of PknB as a key regulator of cell division in streptococci and indicate a profound impact of Carolacton on the coordination between peripheral and septal cell wall growth. The established vector system represents a novel tool to study essential steps of cellular metabolism.

## Introduction

Understanding the biological function of a protein requires knowledge of its cellular localization as exemplified, e.g., by the polar localization of chemotaxis protein clusters and the midcell localization of the divisome complex (Rudner and Losick, 2010; Nevo-Dinur et al., 2012). The correct spatio-temporal distribution of a protein is essential for its biological function (Govindarajan et al., 2012; Nevo-Dinur et al., 2012). Disturbing the localization pattern of essential bacterial proteins is thus a promising strategy for the development of novel antimicrobial substances (Govindarajan et al., 2012).

Carolacton is a small inhibitory molecule active against cells of the caries pathogen *Streptococcus mutans* growing under acidic conditions (Kunze et al., 2010). The strict stereospecific activity at nanomolar concentrations suggests that the substance acts via a completely novel mode of action and has a molecular target which is present in only a few copies per cell (Stumpp et al., 2015). Cell elongation, chain formation and bulging has been observed in cultures treated with Carolacton and suggest defects in cell division and a weakened cell wall (Kunze et al., 2010; Reck et al., 2011; Stumpp et al., 2015). A transcriptome analysis of Carolacton treated cells of *S. mutans* revealed differential expression of genes encoding proteins involved in cell division and the down-regulation of the VicKR two component system controlling cell wall metabolism (Reck et al., 2011). The influence of Carolacton on cell wall metabolism and cell division was further substantiated in a proteome analysis of Carolacton treated biofilms and planktonic cells (Li et al., 2013). Changes in the morphology and defects in daughter cell separation after Carolacton treatment were also observed in other oral bacteria, namely *S. oralis, S. gordonii* and *Aggregatibacter actinomycetemcomitans* (Stumpp et al., 2015). Finally it was shown that the serine/threonine protein kinase PknB is essential for the damage of *S. mutans* cells by Carolacton (Reck et al., 2011). Serine/threonine protein kinases (STPKs) represent master regulators of cell division in streptococci and are thought to mediate the switch from peripheral to septal cell wall growth and vice versa (Beilharz et al., 2012). STPKs containing extracellular C-terminal PASTA (**P**enicillin binding **A**nd **S**erine/**T**hreonine **A**ssociated) domains sense unbound peptidoglycan precursors and ß-lactam antibiotics (Maestro et al., 2011). Upon activation STPKs exert their regulatory function through phosphorylation of target proteins by the intracellular membrane anchored kinase domain. Typical targets of STPKs are proteins involved in translation, peptidoglycan biosynthesis, cell division, control of virulence factors, and resistance against antibiotics and the innate immune system (Pereira et al., 2011). Recently cross-talk between STPKs and two component systems (TCS) has been demonstrated, since response regulators were found to be phosphorylated by STPKs (Pereira et al., 2011).

Despite the obvious influence of Carolacton on cell division it has never been analyzed how it interferes with the divisome and how PknB mediates the deleterious effect of Carolacton on *S. mutans*. Studies evaluating the mode of action of Carolacton were until now entirely based on global approaches and did not take into account differences between individual cells (Reck et al., 2011; Li et al., 2013; Sudhakar et al., 2014). However, Carolacton kills only a subpopulation of biofilm cells in *S. mutans* and phenotypic pleomorphism was also observed in other Carolacton treated bacteria (Kunze et al., 2010; Reck et al., 2011). Therefore we were curious to study the mechanism of Carolacton activity on the single cell level. Disturbing cell wall metabolism and cell division is the mode of action of many known antibiotics, e.g., ß-lactams.

Most of the early studies on the subcellular localization pattern of bacterial proteins used immunostaining which required fixed cells. With the establishment of bright, fast folding and stable GFP variants, fluorescent fusion proteins were successfully applied to track protein dynamics in eukaryotic and bacterial cells on the single cell level *in vivo* (Rizzo et al., 2009a,b). These tools also enable the application of advanced imaging technologies, e.g., time-lapse microscopy (Young et al., 2012). Thus, a high degree of structural organization was detected within the bacterial cell and the previous concept that bacteria contain almost no subcellular structural elements turned out to be false (Rudner and Losick, 2010; Nevo-Dinur et al., 2012). One of the best studied examples of subcellular organization in bacteria is the divisome protein complex, consisting of at least 10 different proteins which are localized at midcell (Typas et al., 2012) and are required for cytokinesis and daughter cell separation. The activity of the divisome is tightly spatio-temporally controlled and linked to chromosome replication and segregation. The PknB homolog STPK and two of its targets, DivIVa and FtsA, were already shown to be part of the divisome complex in *S. pneumoniae* and loss of STPK was demonstrated to completely alter the mechanism how the pneumococcal cell divides (Giefing et al., 2010; Beilharz et al., 2012). The DivIVa protein is highly conserved in Gram-positive bacteria and it is believed to be essential for chromosome segregation and division site selection in cocci (Pinho et al., 2013).

The oral pathogen *S. mutans* represents one of the major contributors to dental caries and it is associated with severe diseases, e.g., infective endocarditis (Nakano et al., 2010). While in other Gram positive and Gram negative bacteria protein localization studies are frequently found in the literature, in streptococci such reports are rare. The study of Eberhardt et al. (2009) is one of the first reports in which the authors established a versatile vector system for the generation of chromosomal GFP reporter strains in *S. pneumoniae*. In *S. mutans* Guo et al. (2013) used the pFW5 suicide vector (Podbielski et al., 1996) to integrate a pH sensitive (pHLuorin) gfp-spaP fusion construct into the *S. mutans* chromosome by single homologous recombination. An inducible expression system which allows stable integration of fusion constructs into the *S. mutans* chromosome via double homologous recombination has not yet been established.

Here the cellular response of Carolacton treatment on cell division is analyzed for first time in individual cells. To this end we constructed fluorescent fusion protein vectors for protein localization studies in *S. mutans*. In these constructs, the expression of the fusion proteins containing an N-terminal GFP+ tag is under the control of various constitutive and inducible promoters. We verified the system by determining the cellular localization of *S. mutans* proteins with known localization in the cell. We compared the different inducible promoters in terms of expression strength and basal transcription and quantified the dose response behavior of the best constructs. Finally we used the reporter system to determine the effect of Carolacton treatment on the localization of the key cell division proteins PknB and DivIVa. Additionally we determined the localization pattern of the unknown proteins SMU_503 and SMU_609. The genes encoding these proteins represent the most strongly upregulated genes upon Carolacton treatment of *S. mutans* biofilms; their expression increases already 5 min after addition of Carolacton (Reck et al., 2011). Using the new molecular tools we gained deeper insights into the mode of action of Carolacton and determined how the viability and cell division of *S. mutans* is disturbed by the substance.

## Materials and methods

### Vector construction

Sequences of primers used for vector construction are deposited in Table S1. Maps of the generated vectors are shown in Figure S1 and a list of the plasmids can be found in Table S2. A list of the resulting *S. mutans* strains after vector transformation is presented in Table 1.

**Table 1:** **Strains used in this study**.

**Strain**	**Genotype**	**Reference**
MR25	UA159::ΔagaL::ΩtetM P_czcD_-gfp+	This study
MR26	UA159::ΔbacA1::ΩtetM P_czcD_-gfp+	This study
MR27	UA159::Δsmu_1405::ΩtetM P_czcD_-gfp+	This study
MR28	UA159::ΔlacE::ΩtetM P_czcD_-gfp+	This study
MR29	UA159::Δsmu_1577ΩtetM P_czcD_-gfp+	This study
MR30	UA159::ΔagaLΩtetM P_gtfB_-gfp+	This study
MR31	UA159::ΔbacA1ΩtetM P_gtfB_-gfp+	This study
MR32	UA159::Δsmu_1405ΩtetM P_gtfB_-gfp+	This study
MR33	UA159::ΔlacEΩtetM P_gtfB_-gfp+	This study
MR34	UA159::Δsmu_1577ΩtetM P_gtfB_-gfp+	This study
MR35	UA159::ΔbacA1ΩtetM P_gtfB_-gfp+-divIVa	This study
MR36	UA159::ΔbacA1ΩtetM P_gtfB_-gfp+-pknB	This study
MR37	UA159::ΔbacA1ΩtetM P_gtfB_-gfp+-vicR	This study
MR38	UA159::ΔbacA1ΩtetM P_gtfB_-gfp+-atpC	This study
MR39	UA159::ΔbacA1ΩtetM P_XylS1_-gfp+-divIVa	This study
MR40	UA159::ΔbacA1ΩtetM P_XylS2_-gfp+-divIVa	This study
MR41	UA159::ΔbacA1ΩtetM P_mutIV_-gfp+-divIVa	This study
MR42	UA159::ΔbacA1ΩtetM P_mutVI_-gfp+-divIVa	This study
MR43	UA159::ΔbacA1ΩtetM P_XylS1_-gfp+-pknB	This study
MR44	UA159::ΔbacA1ΩtetM P_XylS2_-gfp+-pknB	This study
MR45	UA159::ΔbacA1ΩtetM P_mutIV_-gfp+-pknB	This study
MR46	UA159::ΔbacA1ΩtetM P_mutVI_-gfp+-pknB	This study
MR47	UA159::ΔbacA1ΩtetM P_XylS1_-gfp+-smu_503	This study
MR48	UA159::ΔbacA1ΩtetM P_XylS1_-gfp+-smu_609	This study

#### Exchange of *S. pneumoniae* flanks of pJWV25 against homologous flanks of *S. mutans*

*S. pneumoniae* flanks of vector pJWV25 were exchanged against homologous flanks of *S. mutans* in two successive steps to allow chromosomal insertion of reporter plasmids into the *S. mutans* genome via double homologous recombination. For vector construction a recombinase mediated cloning approach was used (Clone EZ kit; Genscript, USA). In the first step vector pJWV25 was PCR amplified using primers P1_EX1/P2_EX1 thereby excising the *bgaA* homologous flank of *S. pneumoniae* from the vector sequence. Downstream flanks of *S. mutans* genes *agaL* (SMU_877), *bacA1* (SMU_1342), SMU_1405, *lacE* (SMU_1491) and SMU_1577 were PCR-amplified using *S. mutans* genomic DNA as template and primers containing 15 bp sequence homology to the PCR amplified pJWV25 vector fragment at their 5′ end. Used primers were D_agaL_For/Rev; D_bacA_For/Rev; D_1405_For/Rev; D_lacA_For/Rev; D_1577_For/Rev. The PCR amplified pJWV25 vector fragment and the respective *S. mutans* flanks were joined using the Clone EZ kit (Genscript, USA) as described previously. Recombinase reactions were transformed in chemical competent *E. coli* DH5α and positive clones were picked and plasmids isolated using the Miniprep Kit (Qiagen, Germany). The correct sequence of the derived plasmids (pMR20-24) was verified by sequencing. Plasmids pMR20-pMR24 were each used as PCR- templates for the second cloning step exchanging the second *S. pneumoniae* flank of pJWV25 (spr0564′) against the *S. mutans* flanks. Primers P1_EX2/P2_EX2 were used to amplifiy vectors pMR20-24. Upstream flanks with *S. mutans* genes were again PCR amplified using the primers listed in Table S1 (U_agaL_For/Rev; U_bacA_For/Rev; U_1405_For/Rev; U_lacA_For/Rev; U_1577_For/Rev). The second cloning steps results in plasmids pMR25-pMR29 carrying upstream and downstream flanks of the genetic loci intended for double homologous insertion into the chromosome (*agaL, bacA1*, SMU_1405, *lacE*, SMU_1577). The plasmid sequence was finally verified by sequencing and plasmids were transformed in *S. mutans* as described before.

#### Exchange of Pczcd promoter against the *S. mutans* GtfB promoter

To construct strains expressing GFP+ fusion proteins under the control of the *S. mutans gtfB* promoter, the *gtfB* promoter sequence was fused to the *gfp*+ gene in a PCR driven overlap extension approach. In the first part of the PCR approach the GtfB promoter and the GFP+ encoding sequence were amplified individually using primer pairs PGtfB_For/Rev and GFP+_For/Rev. Overlapping sequences between both targets were introduced via the 5′ termini of primers PGftB_Rev and GFP+_For. In a second PCR the *gtfB* promoter was fused to *gfp*+ using the amplified products from step 1 as templates (each 10 ng) and primers PGtfB_For /GFP+_Rev for amplification. To finally introduce the GtfB-gfp+ fusion into pMR25-pMR29, the vectors were amplified with primers P1_EX_3/P2_EX_3. The insert was amplified with the primers I_GtfB_GFP+_For/Rev and insert and vector were ligated using the CloenEZ kit as described above. The resulting plasmids pMR30-pMR34 were transformed in *S. mutans*.

#### Construction of pMR31 derived vectors encoding GFP+ fusion proteins under the control of the GtfB promoter

Vector pMR31 (homologous flanks to bacA1, GFP+ under the control of PgtfB) was used as cloning vector for the generation of strains expressing GFP+ fusion proteins. Coding sequences of DivIVa (primers DivIVa_F/R), PknB (primer PknB_F/R), VicR (primer VicR_F/R), atpC (primer atpC_F/R) SMU_503 (primer SMU_503_F/R) and SMU 609 (primer SMU_609_F/R) were thus PCR amplified. Vector pMR31 was amplified with primer pair P1_MR31 and P2_MR31 and the vector was ligated with the purified PCR products of above listed genes. The resulting vectors (pMR35-pMR40) were sequenced and transformed into *S. mutans* UA159.

#### Construction of reporter plasmids with inducible promoters

For the construction of inducible *S. mutans* strains plasmid pMR31 was amplified with primer pair P1Promo/P2Promo thus releasing the GtfB promoter from the plasmid sequence. The promoter sequences of mutacin IV and mutacin VI were amplified from 50 ng of genomic DNA of *S. mutans* UA159 using primer pairs PmutIV_F/R and PmutVI_F/R respectively. For the amplification of the xylose inducible XylS1 and XylS2 cassettes 5 pg of plasmids pZX9 and pZX10 were used as template. Primer pairs XylS1_F/R and XylS2_F/R were used in the PCR reaction to amplify the cassettes. PCR products of the different promoters and the linearized pMR31 vector were purified using the PCR Purification Kit (Qiagen, Germany) and ligated using the Clone EZ approach. The resulting plasmids were verified by sequencing. The coding sequences of the different genes (see above) were subsequently cloned 3′ of gfp+ and the linker sequence, as described above. After transformation of the plasmids into *S. mutans* the generated strains were tested for functionality. Xylose concentrations were 1% and MIP concentration was 2 μM to fully induce gene expression from the promoters.

### Strains and cultivation conditions

All *S. mutans* strains were routinely propagated in in Todd Hewitt broth supplemented with 0.5% (wt/vol.) yeast extract (THBY; Becton Dickinson, Heidelberg, Germany) in an incubator (5% CO_2_, 37°C) without agitation. When indicated, antibiotics were added to the medium (tetracycline 12.5 μg/ml and erythromycin 10 μg/ml). To study the effects of Carolacton a buffered THBY medium was used (75 mM phosphate buffer; pH 6.5).

### Determination of cell length and calculation of fluorescence intensity from microscopic images

The length of Carolacton treated and untreated cells was determined using the Cell Sense Standard Software (Olympus, Germany) measuring 200 individual cells per phase contrast image. Images derived from 3 different regions of each microscopic sample were analyzed and finally the mean of these was calculated.

For the line plots of fluorescence microscopic images the Image J software (http://imagej.nih.gov/ij/) was used. The fluorescence intensity of 5 individual and randomly chosen cells was measured along the main cell axis. The mean of these cells was calculated and the line plots were generated using the Origin 9.0 software (www.originlab.com).

### Flow cytometry

Overnight cultures of the strains grown in THBY were diluted to an OD of 0.1 and grown at 37°C and 5% CO_2_. When the bacteria had reached an OD of 0.2 the culture was divided into two equal fractions. One fraction was induced with either 2 μM synthetic MIP or 1.5% (D)-xylose, the second fraction was used as an uninduced control. Aliquots (0.5 ml) were sampled after 30, 60, 90, 120, 150, and 180 min post induction. Samples were centrifuged (5 min and 7000 rpm) and washed once with PBS. Subsequently the samples were resuspended in 1 ml of ice-cold PBS and sonicated using a MS72 sonotrode with the Sonoplus HD2200 device (Bandelin, Germany) for at least 20 s at 10% power. Settings were a 0.5 s impulse which was followed by a 0.5 s break. Live/Dead staining before and after sonication was performed to exclude that sonication significantly interfered with membrane integrity. For flow cytometry the LSR Fortessa Cell Analyser (BD, Germany) was used. 0.22 μM filtered PBS was applied as sheath fluid. Cytometer settings were chosen as previously reported (Lemme et al., 2011). 50000 cells were analyzed and the resulting data processed with a self-written R-Script.

### Vancomycin bodipy Fl staining

O/N *S. mutans* WT strains were diluted to an OD of 0.1 in buffered THBY (75 mM phosphate buffer; pH 6.5). Cells were divided into two parts, one part was treated with 0.25 μg/ml Carolacton while the other part was used as untreated control. The cultures were grown at 37°C and 5% CO_2_ until an OD_600_ of 0.5 was reached. Aliquots of 100 μl culture were treated with Bodipy Fl vancomycin (Life Technologies, Germany) at a final concentration of 1 μg/ml. Subsequently cells were grown for additional 30 min at 37°C and 5% CO_2_ and then centrifuged at 7000 rpm for 5 min. The supernatant was removed carefully and the cell pellet was washed with 500 μl 1xPBS. Collected cells were analyzed under the fluorescence microscope.

### Fluorescence microscopy

*S. mutans* cultures grown in THBY were centrifuged (7000 rpm; 5 min) and washed two times with 0.85% of NaCl. The bacterial pellet was resuspended in 50-100 μl of NaCl. 3 μl of the re-suspended cells were transferred to a microscopic slide and covered with a cover slide. Fluorescence microscopy was conducted using an Olympus BX60 microscope, equipped with a colorview II camera and a 100x/1.3 oil immersion objective. The filter U-MWIBA3 (excitation, 460–495 nm; emission, 510–550 nm; dichromatic filter, 505 nm) from Olympus (Seelze, Germany) was used to visualize GFP+. Overlay images were generated using the Cell Sense Standard Software (Olympus, Germany). For better visualizations on printouts, brightness and contrast were modified equally for all images using Adobe Photoshop.

## Results

### Construction of vectors for expression of GFP-tagged fusion proteins in *S. mutans*

We modified the system of Eberhardt et al. (2009) for utilization in *S. mutans*. To this end the homologous *S. pneumoniae* flanks were replaced with flanks that allow integration of the vector into the *S. mutans* chromosome. 5 non-essential loci were tested for this purpose; *agaL* (SMU_877), *bacA1*(SMU_1342), SMU_1405, *lacE* (SMU_1491), and SMU_1577. Homologous flanks were cloned into pJWV25 and the resulting plasmids (pMR25-29; Table S2 and Figure S1) were transformed into *S. mutans*. Although a PCR analysis of the genomic DNA of the established strains (MR25-MR29, Table 1) verified the correct integration of the plasmids into the *S. mutans* chromosome via double homologous recombination in all cases, the corresponding zinc inducible strains did not show detectable fluorescence, regardless of the used zinc concentration. The zinc inducible promoter of *S. pneumoniae* might be nonfunctional under the tested conditions, e.g., due to a low import or strong efflux of Zn^2+^ in *S. mutans*. Alternatively, the ortholgoue of the pneumococcal SczA transcriptional regulator (Kloosterman et al., 2007) in *S. mutans* (SMU_439) might not recognize the *S. pneumoniae* promoter sequence.

Thus, the P_czcd_ promoter was replaced with the strong promoter of the glycosyltransferase B gene (*gtfB*) of *S. mutans* (Biswas et al., 2008) in all constructs (plasmids pMR30-34, Table S2). Strikingly all resulting strains (MR30-34, Table 1) showed green fluorescence upon growth in complex medium (Figure S2), indicating that indeed the P_czcd_ promoter is not functional in *S. mutans* under the tested conditions. No significant difference in the growth characteristics or the specific fluorescence was detected between the different strains. Even in prokaryotes the genomic location can have a profound influence on the expression of target proteins (Govindarajan et al., 2012). However, all genomic loci tested here were equally well suited for the expression of GFP+ tagged fusion proteins.

### Subcellular localization of fusion proteins in *S. mutans*

To test whether GFP+ tagged fusion proteins can be successfully expressed with the established vector system and localized in the correct cellular compartment we used pMR31 (integration into the *bacA1* locus) and cloned the coding sequences of different *S. mutans* proteins with cytoplasmic, membrane and midcell/divisome localization into the plasmid (plasmids pMR35-38) and transformed them into *S. mutans*. The resulting *S. mutans* strains MR35-38 were tested for the localization of GFP+ tagged fusion proteins.

**Figure 1:**
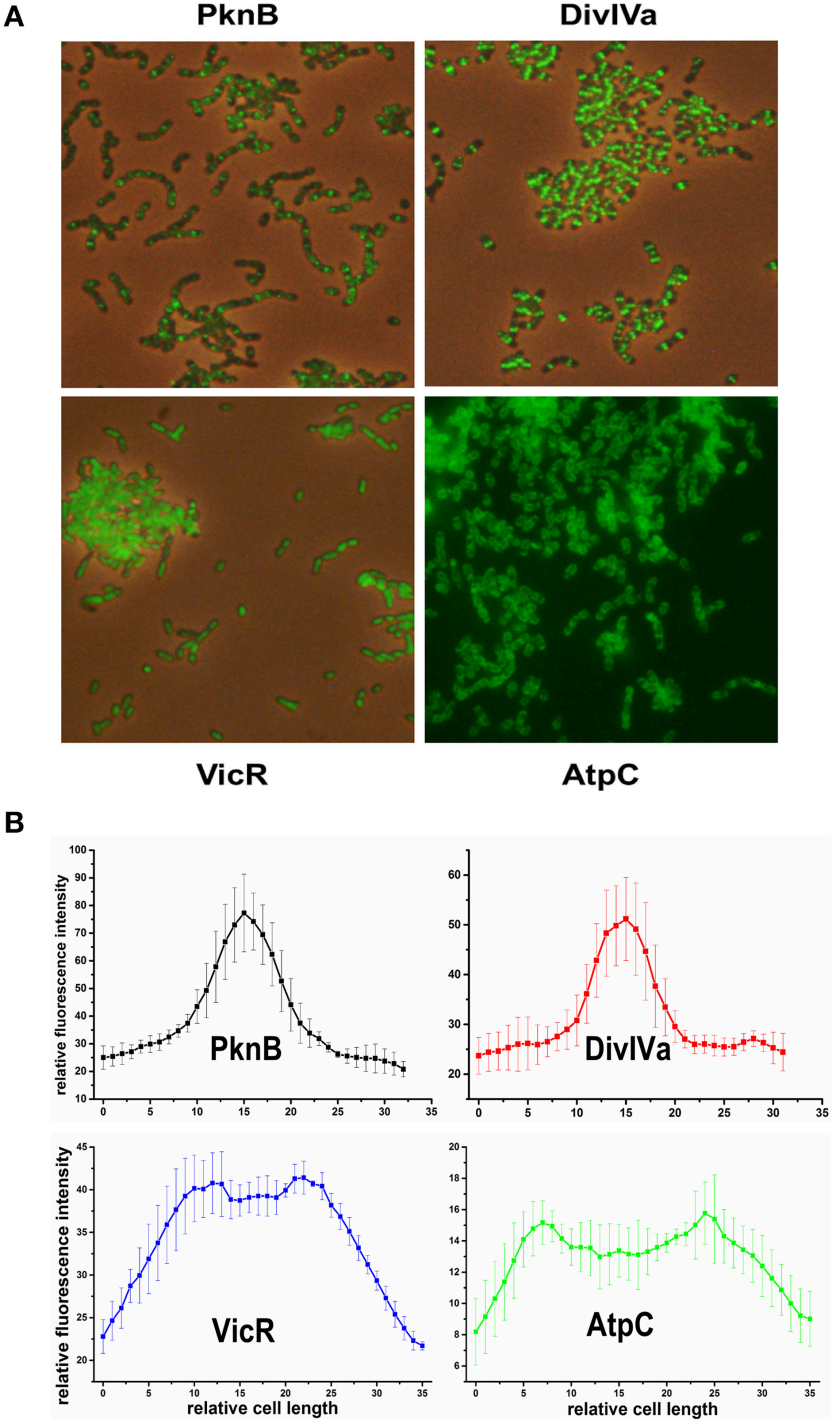
**Localization of PknB-, DivIVa-, VicR-, and AtpC-GFP+ fusion proteins in *S. mutans* UA159**. Expression of the different N-terminally tagged GFP+-fusion proteins in strains MR35-38 is under control of the promoter of the *S. mutans* glucosyltransferase B (*gtfB*). Strains were grown to exponential growth phase (OD_600_ = 0.5) in THBY medium. **(A)** shows overlay images (GFP fluorescence and phase contrast) derived from the PknB, DivIVa, and VicR reporters. Due to the low contrast of the overlay image of strain MR38 a fluorescence image of strain MR38 is depicted. **(B)** shows the fluorescence intensity along the main cell axis of 5 randomly chosen cells. It was determined using the image J software.

We chose PknB and DivIVa because they were shown to be constituents of the divisome in *S. pneumoniae* which is localized at midcell (Beilharz et al., 2012). For cytoplasmatic proteins, we chose VicR, the response regulator of the only essential two component system in *S. mutans* (Senadheera et al., 2005). For proteins localized in the bacterial membrane we chose AtpC, which is part of the proton pumping F1F0 ATPase (Kuhnert et al., 2004). Genes for all of those proteins were genetically fused to *gfp*+ using vector pMR31 yielding the plasmids pMR35-38. Gene expression of the fusion constructs in the resulting strains MR35-38 is under the control of the *S. mutans gtfB* promoter. The localization of these fusion proteins was analyzed in the mid exponential growth phase in complex medium using fluorescence microscopy (Figure 1). Overlay images of the different analyzed strains are shown in Figure 1A while in Figure 1B the fluorescence intensity along the main cell axis of 5 randomly chosen cells is shown.

As expected PknB and DivIVa were clearly localized at midcell at the division site. In Gram positive bacteria fluorescently labeled vancomycin (e.g., Bodipy Fl vancomycin) was frequently applied to visualize cell wall synthesis (Papadimitriou et al., 2007; Raz and Fischetti, 2008; Kashyap et al., 2011). Bodipy Fl Vancomycin binds to uncrosslinked peptidoglycan and thus stains the site of new cell wall synthesis at midcell. A comparison to a *S. mutans* WT strain stained with Bodipy Fl Vancomycin (Figure S3) revealed that both proteins are localized at the site of new cell wall synthesis. Cell wall synthesis in ovoid shaped cocci (ovococci) is exclusively observed at midcell and at the edges of the Z-ring (Pinho et al., 2013). Accordingy the fluorescence intensity (Figure 1B) showed at peak at midcell and resembled a bell-shaped normal distribution.

For the VicR reporter strain MR37 the bacterial cells were found to be entirely fluorescing green, confirming the cytoplasmic localization of GFP+-VicR. Fluorescence intensity of the VicR fusion protein showed a steep increase from the cell poles toward a broad plateau along the main cell axis. This behavior is consistent with the cytoplasmic localization of VicR.

AtpC was localized in the bacterial membrane as expected, too. The GFP fluorescence of strain MR38 was concentrated in a halo surrounding the cytoplasm of the cell. This is clearly reflected in the intensity of fluorescence across the cell axis. Two peaks were observed at the cell poles while the fluorescence intensity in the central region between the cell poles was lower.

To conclude, all studied proteins showed their expected subcellular localization. The established system is therefore applicable for single cell analysis of protein localization in *S. mutans*. In the next step we introduced inducible promoters to be able to investigate timed fusion protein expression in different cellular compartments.

### Establishment of an inducible fluorescent protein expression system in *S. mutans*

Since most native proteins are only transiently expressed, constitutive intracellular overexpression of fluorescent fusion proteins often interferes with host cell metabolism and might cause misfolding of the target protein and/or the fluorescent tag. This is particularly important when studying toxic proteins *in vivo* whose toxicity often depends strongly on their concentration. Thus, utilization of a linearly inducible and tightly controllable expression system is required for studying localization and dynamics of folding-sensitive and toxic proteins. In *S. mutans* only very few inducible expression systems are presently available as genetic tools and most of them show either low induction ratios and/or high basal transcription. Xie et al. developed two fine-tunable xylose inducible promoter cassettes and demonstrated their application by studying toxin/antitoxin systems in *S. mutans* (Xie et al., 2013). The XylS1cassette is specifically designed for high expression levels at the cost of higher basal transcription, while the XylS2 cassette ensures very low basal transcription but only medium expression strength (Xie et al., 2013). Thus, reporter strains expressing the GFP+-DivIVa and GFP+-PknB proteins under the control of either the XylS1or the XylS2 promoter cassettes were generated. To this end plasmids pMR35 and pMR36 were modified and the *gtfB* promoter was replaced by either the XylS1 or the XylS2 promoter cassette yielding plasmids pMR39-40 (DivIVa) and pMR43-44 (PknB). In addition we constructed strains whose GFP+ fusion protein expression is inducible by the mutacin inducing peptide (MIP), formerly termed competence stimulating peptide (CSP). We previously demonstrated that MIP highly induces expression of bacteriocin encoding genes independent from the growth medium (Perry et al., 2009; Reck et al., 2015), including the two peptide bacteriocin Mutacin IV (SMU_150 and SMU_151) and Mutacin VI (SMU_423). Bacteriocin production was shown to be tightly regulated in *S. mutans*, thus making MIP responsive promoters potentially effective gene induction systems (Perry et al., 2009; Reck et al., 2015). The promoters of mutacin IV and mutacin VI were cloned 5′ of the GFP+ coding region of plasmids pMR35 and pMR36, thereby replacing the *gtfB* promoter. After transformation of the resulting plasmids pMR41-42 and pMR45-46 in *S. mutans* the MIP inducible strains MR41-42 and MR45-46 expressing DivIVa and PknB GFP+ fusion proteins were obtained.

**Figure 2:**
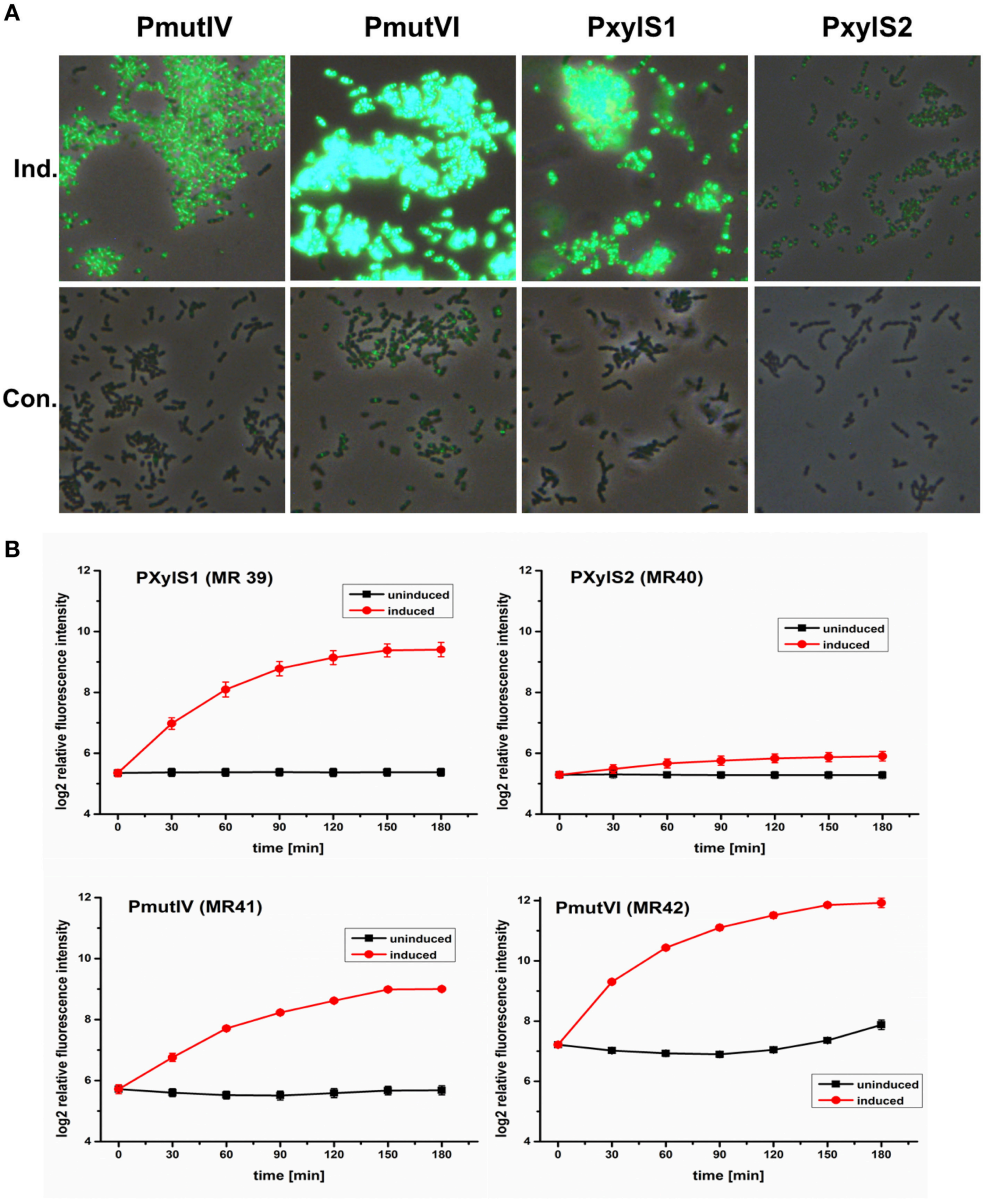
**Comparison of the expression strength and basal transcription of different inducible GFP+-DivIVa reporter strains in *S. mutans* UA159**. D-xylose inducible strains carrying a chromosomal *gfp*+*-divIVa* fusion construct under the control of the XylS1 (MR39) and XylS2 (MR40) promoter cassettes, respectively, were constructed in *S. mutans*. In addition DivIVa reporter strains inducible by the competence stimulating peptide (MIP) and carrying the mutacin IV (PmutIV, MR41) and mutacin VI promoter (PmutVI, MR42) were generated. Strains MR39-42 were grown in complex THBY medium to early exponential growth phase (OD_600_ = 0.2) and GFP+-DivIVa expression was fully induced with either 2 μM MIP (pMR41-42) or 1.5% (D)-Xylose (MR39-40). 0, 30, 60, 90, 120, 150, and 180 min post induction cells were collected, washed and analyzed using fluorescence microscopy. In **(A)** overlay microscopic images (green fluorescence/phase contrast) of the corresponding induced (upper panel) and uninduced strains (lower panel) 3 h post induction are presented. In part **(B)** line plots of the relative fluorescence intensity in course of time of strains MR39-42 are shown. For the generation of line plots the GFP fluorescence of 50,000 individual cells was recorded using flow cytometry. Red line plots indicate induced strains, while in the black line plots the fluorescence intensity of the corresponding uninduced strains is shown. The mean and the standard deviation of three independent biological replicates are presented.

All four inducible DivIVa expression strains (MR39-42) showed maximal DivIVa expression using MIP or xylose (2.5 μM and 1.5% respectively) for induction. Figure 2A shows that expression of DivIVa was strongest from the mutacin VI promoter, followed by the XylS1 promoter and the mutacin IV promoter. The weakest DivIVa expression was observed from the XylS2 cassette. However, for the strains carrying the mutacin IV and VI promoter constructs (MR41 and MR42) DivIVa-GFP fluorescence was observed also in uninduced samples, likely caused by endogenous MIP production. Thus, application of these reporters is restricted to conditions under which MIP is not strongly endogenously produced. Alternatively the MIP encoding gene *comC* could be deleted. However, it also has to be considered that the MIP inducer interferes with *S. mutans* physiology due to the induced expression of various bacteriocins (Perry et al., 2009). For the xylose inducible strains MR39 and MR40 no DivIVa-GFP expression was observed in the uninduced controls, indicating their excellent applicability as inducible expression systems.

We then quantified the development of DivIVa-GFP fluorescence during growth using flow cytometry. Strain MR42, carrying the mutacin VI promoter, represented the strain that was most strongly inducible. The mean GFP fluorescence increased approximately 5 orders of log2 intensity during 180 min compared to the level prior to induction. However, at 180 min the uninduced control of this strain also showed a significant increase in fluorescence intensity, likely due to endogenous MIP production. The basic fluorescence level of the uninduced strain MR42 was considerably higher than that of strains MR39-41, indicating a higher basal transcription from the mutacin VI promoter than from the mutacin IV promoter.

Compared to MR42 the second MIP responsive strain MR41 was weaker inducible and less bright. Nevertheless induction increased the fluorescence by 3 orders of log2 intensity above the background level. The fluorescence of the uninduced strain did not increase significantly during growth, indicating that the mutacin IV promoter might be less sensitive to endogenously produced MIP. The xylose inducible strains MR39 and MR40 showed a sigmoidal increase in GFP fluorescence during growth and reached their maximum 180 min post induction. Both strain showed a comparable background level of low GFP fluorescence in the uninduced controls. However, strain MR39, carrying the XylS1 promoter cassette, was much stronger inducible (4 orders of log2 intensity) than strain MR40 carrying the XylS2 cassette.These findings are in accordance with the report of Xie et al. (2013). The authors demonstrated that the expression level of a luciferase gene was much higher for a XylS1 promoter construct than for a XylS2 promoter construct. Xie et al. also found that the XylS2 controlled strain has a lower basal expression level than the XylS1 carrying strain (Xie et al., 2013). This was not observed in our analysis and might be due to the higher detection limit of GFP compared to luciferase.

To compare the applicability of the constitutive reporter strains for protein localization studies with that of inducible strains we quantified the GFP fluorescence of two strains (MR35 and MR49) constitutively expressing a DivIVa-GFP+ fusion during growth (Figure S4). DivIVa expression in the strain MR49 is under control of the strong lactococcal P23 promoter (Biswas et al., 2008) while strain MR35 carries the *gtfB* promoter. The non-fluorescent *S. mutans* WT strain was used to determine the autofluorescence (Figure S4). In comparison to the inducible expression strains MR39 and MR41-42 (see Figure 2) the fluorescence of strains MR35 and MR49 was relatively weak (Figure S4). Thus, the inducible expression strains are superior for protein localization studies. To conclude, the high basal transcription of the mutacin VI promoter (MR 42) and its strong response to endogenously produced MIP make it unsuitable for expression studies. The promoter of mutacin IV is too weakly induced to generate clear signals. The XylS1 promoter (MR39) is well suitable, although it is slightly less inducible than the Mutacin VI promoter. Thus, we subsequently used XylS1 based expression constructs which combine a low basal transcription with a high level of induction.

For this construct (strain MR39) we then quantified the dose-response behavior to the inducer D-xylose for concentrations ranging from 0.005% (3.33^*^10^3^ μM) to 4% (2.66 ^*^10^5^ μM). GFP expression was determined 3 h after induction which represents the peak of fluorescence (Figure 2B). The dose response curve had a sigmoid shape (Figure S5A), concentrations above 0.1% (6.66^*^10^3^ μM) strongly induced GFP fluorescence, while the response was saturated at concentrations exceeding 2% (1.33^*^10^5^ μM) D-xylose. Between 0.2 and 2% the response was linear and thus these concentrations are suitable to obtain a range of expression levels of the fusion proteins. The EC_50_ value was calculated to be 2.86^*^10^4^ (±0.51^*^10^4^) μM D-xylose (Figure S5B).

### Effect of carolacton on the localization pattern of cell division proteins

**Figure 3:**
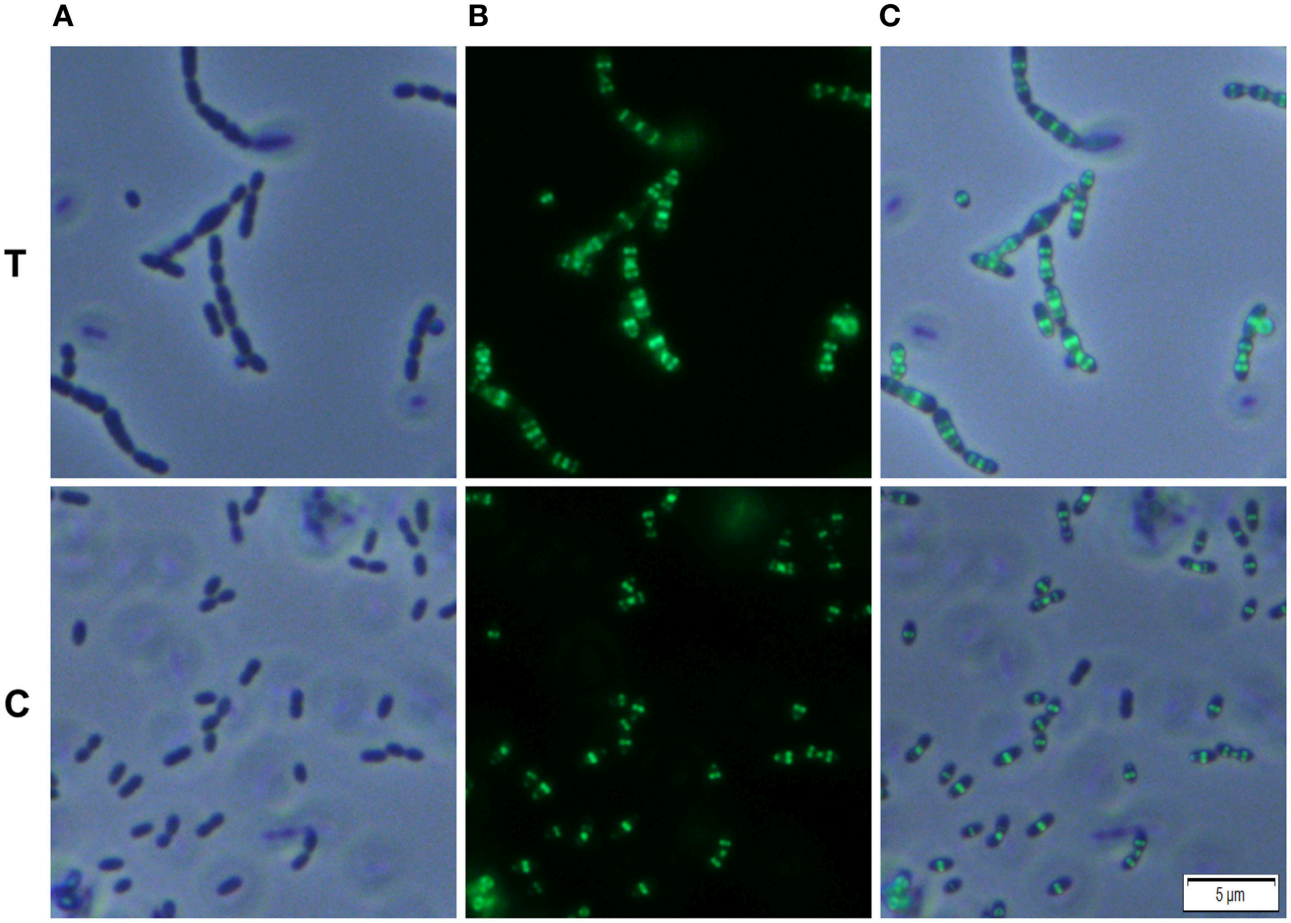
**Effect of Carolacton treatment on the localization of the cell division protein DivIVa in *S. mutans* UA159**. The chromosomal GFP+-DivIVa reporter strain MR39 was grown in buffered (75 mM and pH 6.5) complex THBY medium to the early exponential growth phase (OD_600_ = 0.2). Cells were treated with and without 5.3 μM Carolacton and GFP+-DivIV expression was induced for all samples (treated/untreated) with 1.5% D-xylose. 3 h post induction cells were harvested, washed and analyzed under the fluorescence microscope. Phase contrast (column **A**), fluorescence (column **B**) and overlay images (column **C**) of Carolacton treated (upper panel **T**) and untreated control cells (lower panel **C**) are presented.

**Figure 4:**
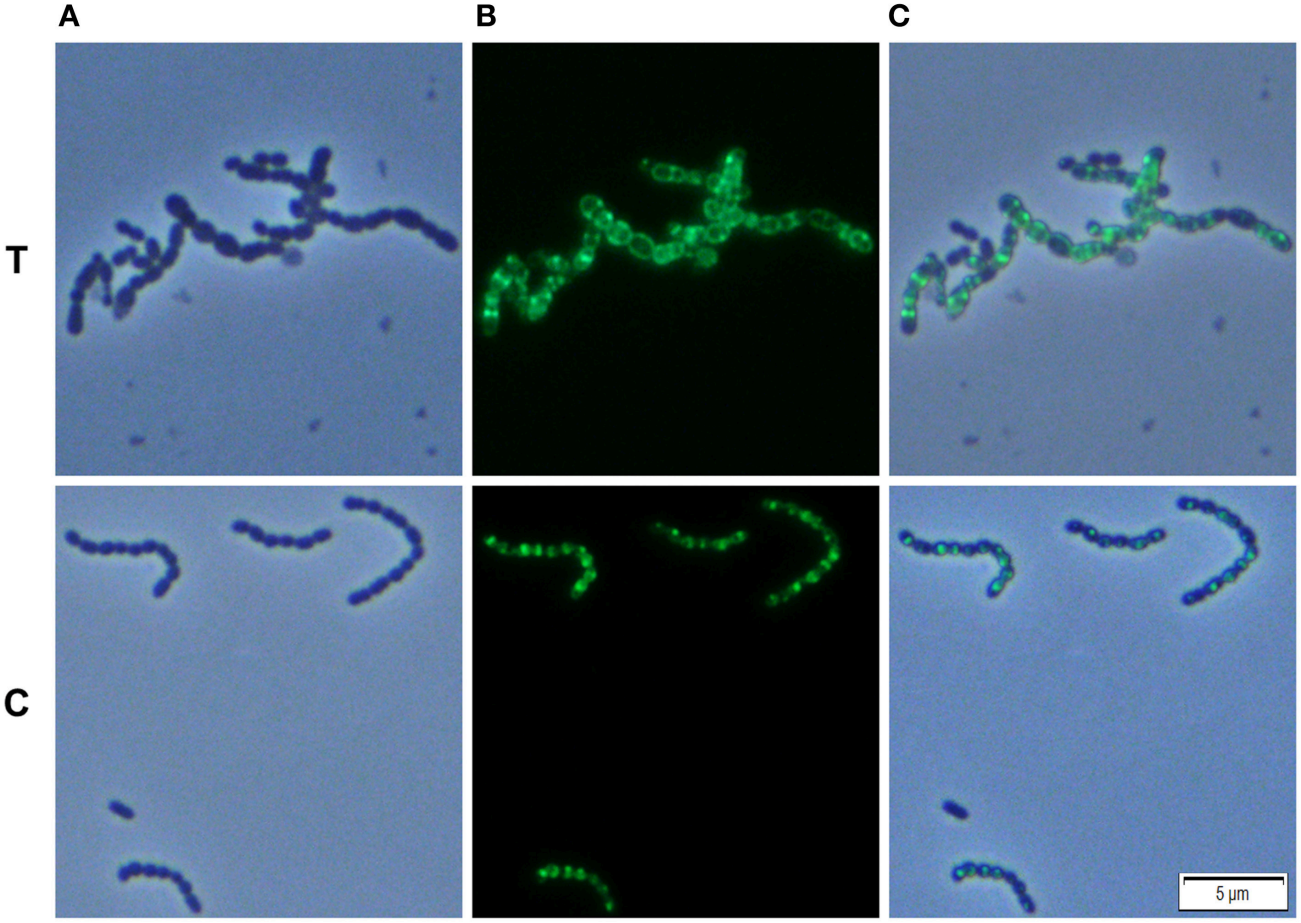
**Effect of Carolacton treatment on the localization of the cell division protein PknB in *S. mutans* UA159**. The chromosomal (D)-xylose inducible GFP+-PknB reporter strain MR43 was grown in buffered (75 mM and pH 6.5) complex THBY medium to the early exponential growth phase (OD_600_ = 0.2). Cells were treated with and without 5.3 μM Carolacton and GFP+-PknB expression was induced for all samples (treated/untreated) with 1.5% D-xylose. 3 h post induction cells were harvested, washed and analyzed under the fluorescence microscope. Phase contrast (column **A**), fluorescence (column **B**), and overlay images (column **C**) of Carolacton treated (upper panel **T**) and untreated control cells (lower panel **C**) are presented.

We analyzed whether Carolacton alters the localization pattern of the cell division proteins PknB and DivIVa. Thus, the xylose-induced (1.5%) reporter strains MR39 and MR43, expressing GFP fusions of DivIVa and PknB respectively, were grown to mid-exponential phase under buffered acidic conditions (pH 6.5, 75 mM phosphate buffer) in the presence of Carolacton. The acidic pH is a prerequisite for the membrane damage and reduction in viability caused by Carolacton in *S. mutans* (Reck et al., 2011). Carolacton treatment caused aberrant cell morphologies with strongly elongated and enlarged cells and the formation of long chains (Figure 3). The mean length of *S. mutans* single cells increased from 1.02 μm (±0.21 μm) to 1.32 μm (±0.47 μm) and was more variable, as indicated by the higher standard deviation. However, “normal” cells remained in the Carolacton treated samples, demonstrating that phenotypic heterogeneity makes some cells more sensitive than others. For both strains Carolacton treatment caused an increase in septum formation (Figures 3, 4). Some cells contained 3 septa per cell, indicating impaired septal closure and thus lack of correct daughter cell separation. In the Carolacton treated samples of strain MR39 (Figure 3, upper panel) DivIVa was localized simultaneously at midcell and at the new cell poles, while it was found mainly at midcell in the untreated controls, again suggesting that septal closure is proceeding too slow to properly constrict the dividing cell. The appearance of extremely large and elongated cells might be caused by inhibition of the septal cell wall synthesis machinery. Thus, peripheral cell wall synthesis elongated the cell, while the correct onset of septal cell wall synthesis and thus septum closure to constrict the daughter cells failed. Thus, the occurrence of elongated cells and cell chains could both be explained by this mechanism. As PknB is known to control switching between peripheral to septal cell wall synthesis (Beilharz et al., 2012), we analyzed the effect of Carolacton on PknB localization using strain MR43. A significantly altered localization pattern of PknB in comparison to the untreated control was observed (Figure 4). While in the controls PknB was located exclusively at midcell as expected, it was dispersed over a much larger part of the cell in Carolacton treated cultures. In some cells PknB even seemed to be randomly distributed in the bacterial membrane, indicated by a halo of green fluorescence around the cells. The observed influence of Carolacton treatment on PknB localization is in accordance with the proposed mode of action of Carolacton via PknB (Reck et al., 2011). These results demonstrate that Carolacton has a profound impact on cell division in *S. mutans* and causes partial delocalization of the key regulator of cell division PknB. The inhibition of septal cell wall synthesis might be a mechanistic consequence of this delocalization. Moreover these results demonstrate the importance of PknB as a main regulator of cell division in streptococci.

In the xylose-induced but non-Carolacton treated strain MR43 slight cell chaining was observed (Figure 3), which was not observed in the corresponding uninduced controls. To exclude that the Carolacton caused delocalization of cell division proteins is an artifact of xylose induction by the XylS1 promoter, we repeated the experiment using strains MR40 and MR44 which carry the XylS2 promoter. In these strains the expression level of the PknB and DivIVa fusion proteins is significantly lower, thus the probability that the fusion protein expression interferes with the *S. mutans* metabolism is lower. We found the same altered patterns of cell division and delocalization of the cell division proteins PknB and DivIVa for the Carolacton treated samples as with the XylS1 promoter construct (Figures S6, S7). Moreover, no chaining was seen in the xylose induced but non-Carolacton treated strain MR44. Thus, higher expression levels of PknB should be avoided as they interfere with the physiology and cell division of *S. mutans*.

It has to be taken into account that the fluorescent tag can alter the localization pattern of the studied protein (Margolin, 2012). Therefore we used Bodipy-FL vancomycin staining to verify the Carolacton-mediated alterations in cell division observed in the reporter strain analysis. Fluorescent vancomycin staining targets the sites of nascent cell wall synthesis and was frequently applied to study cell division in Gram-positive bacteria (Papadimitriou et al., 2007; Raz and Fischetti, 2008). In the untreated controls most of the stained cells had a single equatorial fluorescent band, thus new cell wall was synthesized at the division site at midcell, in accordance with the proposed model of cell wall synthesis in ovoid cocci (Pinho et al., 2013). Some cells synthesized new cell walls simultaneously at the old and the new division site, thus both the equator and the future poles of the dividing cell were fluorescing green. This might indicate that peripheral cell wall synthesis exceeded the septal cell wall synthesis in these cells. Thus, although the constriction of the two daughter cells had not been finished properly the peripheral cell wall machinery already started elongating the new cells.

Carolacton treated cells showed increased septum formation and a significantly altered pattern of cell wall synthesis. In full accordance with the findings using the DivIVa and PknB GFP+ reporter strains, the fluorescent dye was more randomly distributed throughout the cell surface than in the controls, and for some cells the whole membrane or the entire cell surface was fluorescing, confirming the profound disturbance of cell wall synthesis and septum placement in Carolacton treated cells.

### Localization of the hypothetical protein SMU_503 and the autolysin SMU_609

**Figure 5:**
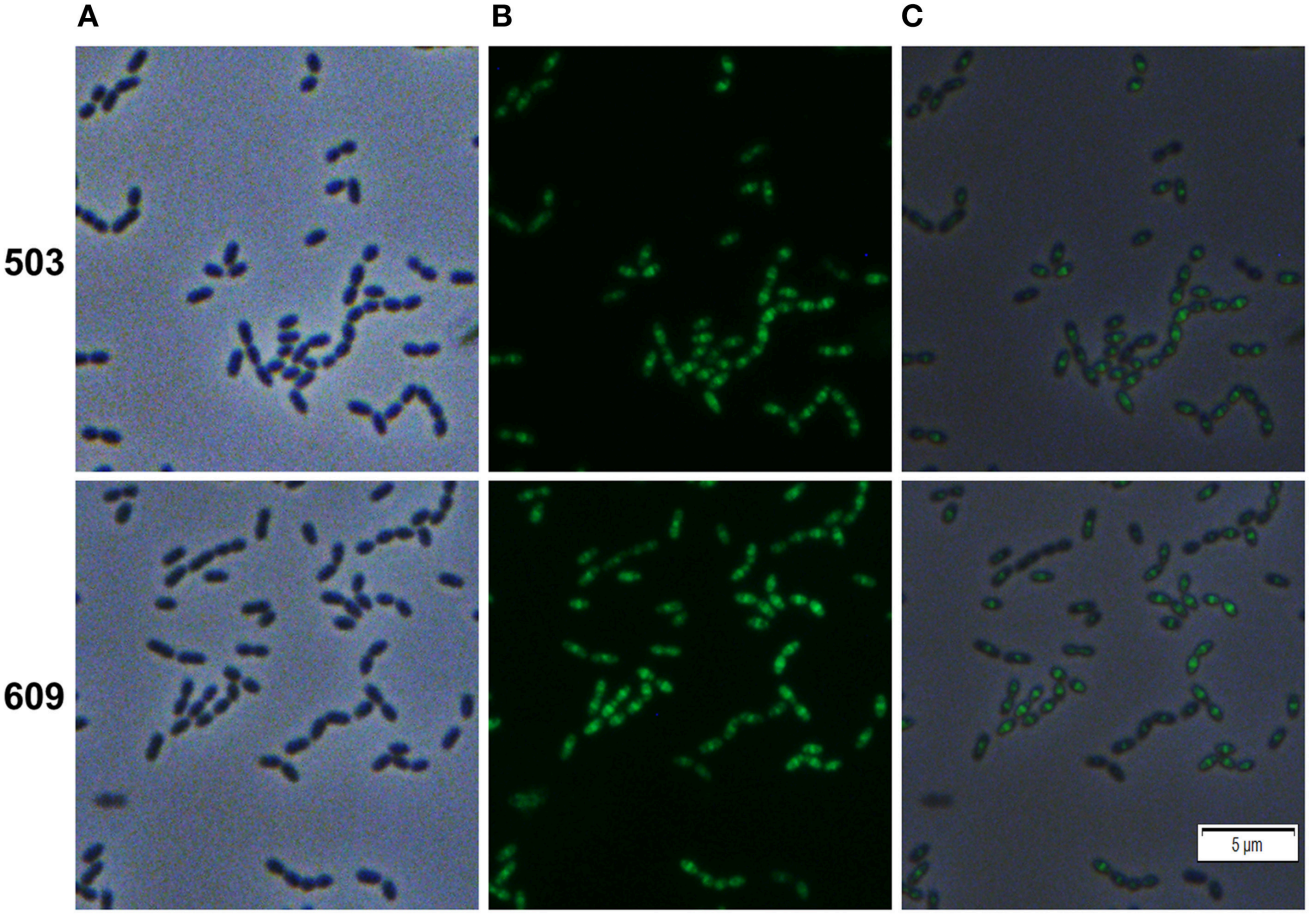
**Cellular localization of the hypothetical proteins SMU_503c and SMU_609 in *S. mutans* UA159**. Xylose inducible reporter strains MR47 (SMU_503c) and MR48 (SMU_609) carrying GFP+ fusions of the genes under the control of the XylS1 promoter cassette were grown in complex THBY medium. Upon entering the exponential growth phase (OD_600_ = 0.25) strains were induced with 1% xylose and cultivated for further 3 h. Cells were harvested, washed and analyzed using fluorescence microscopy. Phase contrast (column **A**), fluorescence (column **B**) and overlay images (column **C**) are presented. In the upper panel **(503)** the images derived from strain MR47 are presented, while the lower panel **(609)** shows the images of strain MR48.

The genes SMU_503 and SMU_609 were both strongly and instantaneously upregulated upon treatment of *S. mutans* biofilm cells with Carolacton. Both genes are coexpressed and share a high degree of sequence similarity in their 5′ UTR sequence (Reck et al., 2011). A bioinfomatic analysis using the TMHMM (http://www.cbs.dtu.dk/services/TMHMM/) membrane domain prediction server discovered that both proteins contain one transmembrane helix in close vicinity to their N-terminus (Figure S8). While the N-terminus of the proteins is predicted to be localized in the cytoplasm, the larger C-terminal part of the protein is likely localized outside the cell. The analysis suggests that both proteins are located in the membrane of *S. mutans*. For SMU_609 a murein hydrolase activity was experimentally verified, indicating that the protein acts as an autolysin upon cell wall synthesis and might thus be involved in cell division (Catt and Gregory, 2005). To study the localization pattern of the two proteins, their coding sequences were cloned into the vector pMR39 thereby replacing the DivIVa coding sequence and xylose-inducible reporter strains were generated (strain MR47 and MR 48 respectively). Figure 5 shows that both SMU_503 and SMU_609 are located at midcell, likely at the place of septal formation. Thus, it is likely that both proteins are involved in cell division.

## Discussion

We established a suite of vectors for expression of GFP-fusion proteins in the oral pathogen *S. mutans* and applied it to elucidate the disturbance of cell division caused by the small molecule inhibitor Carolacton.

Maturation of GFP strictly requires the presence of oxygen (Shaner et al., 2005). Thus, the microaerophilic or anaerobic conditions under which streptococci are cultivated might have limited the application of fluorescent reporter strains for them so far. However, as proof of concept Veening *et al*. used the very bright GFP+ protein to establish a chromosomal reporter strain system in *S. pneumoniae* which is applicable for single cell investigations. Here we used a similar approach to establish a GFP+ based reporter strain system in the oral pathogen *S. mutans*. In a previous study we had observed slow fluorophore maturation for longer wavelength GFP variants (DsRed like chromophores) in *S. mutans* (Reck et al., 2015). Thus, application of these fluorescent proteins for single cell reporter strains is not feasible. GFP+ represents one of the brightest GFP variants presently available, it was estimated that its brightness is 130 fold higher than that of wild-type GFP (Scholz et al., 2000). Thus, the constraint of poor fluorophore maturation can be overcome by using bright GFP variants that produce detectable signals even if only a subfraction of the synthesized GFP molecules mature properly and develop fluorescence. The use of fluorescent proteins that do not require oxygen for maturation (e.g., flavin mononucleotide based fluorescent proteins) is hampered by their low intrinsic brightness (Drepper et al., 2007).

Identical to the system of Eberhardt et al. (2009) our vectors allow stable chromosomal integration of the genetic GFP+ fusions via double homologous recombination at a non-essential locus. Four different inducible promoters with significantly different promoter strength allow protein expression over a wide range of cellular concentrations in our strains. In contrast, the system of Eberhardt et al. is based solely on the zinc inducible PczcD promoter. Higher concentrations of Zn^2+^ are toxic for the cell (Djoko et al., 2015; Ong et al., 2015) which limits the possible inducer concentrations and thus the dynamic range of fusion protein expression. Using our xylose inducible reporter strains such toxic effects are unlikely. Moreover, the more than 600 fold increase in reporter activity observed for the xylose inducible cassettes (Xie et al., 2013) was considerably higher than the 22 fold increase reported for the zinc inducible promoter (Kloosterman et al., 2007). However, a disadvantage of the utilization of sugar-inducible promoters is the potential metabolism of the inducer by the cell.

Studying the cellular localization and dynamics of a protein in its native environment with the help of FP tagging requires individual modulation of the expression levels of the fusion protein. The expression of the FP-fusion must be high enough to generate a fluorescence signal above the background autofluorescence while *vice versa* higher expression levels might disturb the native localization pattern, interfere with host metabolisms and might result in misfolding of the protein of interest (Snapp, 2005). Using e.g., the xylose induced strain MR43 expressing GFP+-PknB under the control of the strong XylS1 cassette, we observed cell chaining and slightly elongated cells, a feature which was not observed in the uninduced control or in the corresponding XylS2 controlled and xylose induced strain MR44. This indicates that the stronger expression of PknB from the XylS1 promoter cassette alters cell division and represents a bias caused by the reporter strain. Thus, high expression rates of PknB should be avoided. This emphasizes the need for inducible expression systems that allow fine-tuning of protein expression to optimize the expression level for each fusion protein individually.

However, independent of the level of expression of a fusion protein, a fluorescent tag can alter the localization pattern of the protein of interest (Margolin, 2012). For the actin-like cell division protein MreB it was shown by cryo-electron tomography that the observed helical structure of a MreB-YFP fusion protein in *E. coli* was an artifact of the fluorescent tag and was not observed for the untagged protein (Swulius and Jensen, 2012). The native protein is localized in dynamic patches. It was postulated that the fluorescent tag introduced at the N terminus of the protein disrupts the N-terminal amphipathic helix which is involved in tethering the cytoplasmic MreB protein to the membrane (Margolin, 2012). Instead of binding to the membrane N-terminal tagged MreB tends to self-aggregate and form long filaments, resulting in the observed helical structures. Thus, it seems advisable to verify the observed localization pattern of a protein by independent methods. Here we used Bodipy-Fl vancomycin staining to analyse the effect of Carolacton on cell division with an independent approach. Our observations made by the DivIVa and PknB reporter strain analysis were fully supported using this independent experimental approach, namely increased septum formation and a delocalization of cell wall synthesis sites upon cell division. The midcell localization of GFP+ fused PknB and DivIVa proteins, as observed in our reporter strains, clearly indicates that these proteins are biologically active and correctly localized.

For ovoid cocci like *S. mutans* a two-state model of peptidoglycan biosynthesis was developed explaining cell elongation and division with one peripheral and one septal mode of cell wall synthesis (Pinho et al., 2013). It is likely that the different modes of cell wall synthesis involve two different machineries, one dedicated to cell elongation and another responsible for septum formation and constriction (Pinho et al., 2013). Cell elongation and the rod-like morphology of Carolacton treated cells are indicative for a severe imbalance between septal and peripheral cell wall synthesis. According to the two-state model of cell wall synthesis mentioned above, the elongated phenotype of Carolacton treated cells can be interpreted as an inhibition of septal cell wall synthesis. Thus, the peripheral cell wall synthesis exceeds septal cell wall growth. Increased septum formation may be observed because septal cell wall synthesis is too slow for proper septum closure. As an alternative the proteins guiding proper Z-Ring placement might be incorrectly localized after Carolacton treatment.

Furthermore, it was shown recently that STPKs control switching between peripheral and septal cell wall synthesis to ensure proper Z-Ring closure and daughter cell separation (Beilharz et al., 2012). Beilharz et al. demonstrated that a STPK deletion strain has a significantly altered mechanism of cell division, leading to elongated cells with multiple, often unconstricted Z-Rings (Beilharz et al., 2012). The correct timing of septal cell wall synthesis is lost in these mutants, instead the division site is split into 2 or more rings and a lateral cell wall elongation occurs between the rings leading to elongated cells (Beilharz et al., 2012). Carolacton treated *S. mutans* cells show a comparable phenotype, indicating that PknB likely mediates the Carolacton caused effect on cell division and cell wall synthesis as reported previously (Reck et al., 2011). Accordingly in our analysis the GFP+- PknB fusion was found to be delocalized upon Carolacton treatment. Thus, due to the interference of Carolacton with the key coordinator of the two cell division modes in ovoid cocci, cell wall homeostasis and morphology of *S. mutans* is severely disturbed. In this context the *pknB* dependency of the Carolacton caused cell death phenotype (Reck et al., 2011) might also be explained. Disturbance of cell wall metabolisms and cell division was often shown to result in a lethal phenotype for the cell (Typas et al., 2012).

The increased septum formation of Carolacton treated cells might be indicative of a lack of coordination between chromosome segregation and septal cell wall synthesis. The directionality of chromosome segregation was already shown to determine the placement of the division plane. The DivIVa protein is a direct target of PKnB and is thought to be mainly responsible for the correct septum placement after chromosome segregation (Pinho et al., 2013). Thus, the failed septum placement and closure observed upon Carolacton treatment is in full accordance with the PknB dependent mode of action of Carolacton and the altered DivIVa localization pattern observed upon Carolacton treatment. However, the DivIVa delocalization was less pronounced than that observed for the PknB protein. Again this is indicative that PknB rather than DivIVa localization is disturbed by the substance. It might be speculated that DivIVa is correctly localized but not properly phosphorylated due to the Carolacton mediated disturbance of PknB activity. Thus, the protein is present at it intended cellular site but might lack its biological activity. It remains to be elucidated whether the correct localization of DivIVa is dependent on the presence of PknB. Recently it was discovered in *S. pneumonaie* that the protein MapZ represents another STPK target and might also function in division site selection (Fleurie et al., 2014). Whether Carolacton treatment also interferes with the localization of the MapZ ortholog in *S. mutans* is another interesting question which needs to be studied in the future.

Here we identified the so far largely uncharacterized proteins SMU_503 and SMU_609 to be localized at midcell and thus being likely components of the divisome machinery. The genes encoding these proteins were instantaneously upregulated upon Carolacton treatment and thus belong to the primary cellular response (Sudhakar et al., 2014). Deletion of these genes did not alter the sensitivity of *S. mutans* toward Carolacton, demonstrating that they are not essential for the mode of action of Carolacton (Reck et al., 2011; Sudhakar et al., 2014). Therefore, their upregulation most likely represents a compensation mechanism of the cell to cope with the deleterious effects of Carolacton.

For SMU_609 an autolysin activity was verified (Catt and Gregory, 2005). Thus, it is highly likely that the protein is involved in cell wall remodeling upon cell division. For both, lateral and septal cell wall synthesis, a tightly controlled and balanced interplay between new cell wall synthesis and breakdown of the old cell wall to insert new peptidoglycan precursors must exist (Typas et al., 2012). Disturbing homeostasis between cell wall synthesis and cell wall breakdown is likely lethal for the cell. As both the peripheral and septal cell wall machineries are localized at midcell in ovoid cocci (Pinho et al., 2013), it is hard to predict whether SMU_609 is involved in the hydrolysis of lateral or septal cell wall. Based on the observation that mainly septal cell wall synthesis is inhibited by Carolacton treatment (elongated cells), it can be speculated that the upregulation of SMU_609 found after Carolacton treatment (Reck et al., 2011; Li et al., 2013) is a compensation reaction of the cell. Consequently SMU_609 might belong to the septal cell wall machinery.

For SMU_503 a Pfam database analysis showed that the protein contains several eukaryotic-like domains with unknown functions. Due to the presence of a typical folate carrier domain the protein may function in the import of folate into the cell and thus might be particularly involved in purine, pyrimidine and methionine metabolism. Accordingly the key genes of pyrimindine metabolism were instantaneously upregulated together with SMU_503 in Carolacton treated cells (Sudhakar et al., 2014). Bacteria are often dependent on a *de novo* biosynthesis pathway for folate. Bacterial enzymes of this pathway are attractive drug targets due to their complete absence in mammals (Bermingham and Derrick, 2002). Firmicutes are able to both synthesize and import folate. Genes encoding high affinity folate binding proteins were found in many Firmicutes and are sometimes localized adjacent to folate salvage genes (Eudes et al., 2008). Folate is particularly important in periods of rapid cell division, thus the co-localization of SMU_503 with the divisome might be explained in this context. The SID-1 RNA_chan and the Serinc domain indicate that SMU_503 is localized in the membrane and may also function as a dsRNA channel.

Taken together, the observations made in this study demonstrate the versatile nature of GFP based localization reporter strains to study the effect of antimicrobials on cellular metabolism. The utilization of single cell fluorescent reporter strains to elucidate the mode of action of antimicrobials was e.g., applied by Beilharz et al. (2012). The authors found that antibiotics targeting the last steps of cell wall synthesis cause a GFP+-STPK delocalization which further indicates a regulatory role for STPKs in cell wall metabolism. Using a MinD-GFP fusion Wenzel *et al*. elucidate that small cationic antimicrobial peptides delocalize peripheral membrane proteins with strong implications on the membrane potential and the respiration in *B. subtilis* (Wenzel et al., 2014). The same authors demonstrated earlier that the delocalization of GFP-MinD fusion proteins represents an indicator for antimicrobials disturbing the membrane potential. With the help of FP reporter strains it thus might also be promising to screen compound libraries for substances that alter the localization pattern of essential virulence proteins but do not kill the bacteria. Compounds that attenuate virulence are unlikely to cause resistance development and might represent a fruitful future alternative for antibiotics (Keyser et al., 2008).

Moreover our study shows that STPK represent attractive drug targets due to their central regulatory role in virulence, host-cell interaction, cell division and cell wall synthesis (Pereira et al., 2011). Thus, approaches targeting STPKs or their cognate phosphatases gain increasing attention in drug discovery.

Fluorescent fusions proteins dramatically changed our perspective on bacteria which were previously considered as simple, unstructured vessels filled with nutrients and enzymes. The surprisingly high degree of structural organization within the well-studied organisms *E. coli* and *B. subtilis* implies that a wealth of novel mechanisms of protein localization waits to be discovered also in streptococci. Our established system can be applied to study cell division in *S. mutans* in general. Determining the localization of key cell division proteins in different gene deletion background can provide valuable information which factors are responsible for the correct localization and assembly of the cell division machinery. Understanding protein localization and dynamics in more detail will provide new tools and strategies to fight back bacterial infections.

## Author contributions

MR designed and conducted the experiments, MR and IW designed the study and wrote the manuscript.

## Funding

MR was funded by the BMBF (program e:bio; grant number 031 A299).

### Conflict of interest statement

The authors declare that the research was conducted in the absence of any commercial or financial relationships that could be construed as a potential conflict of interest.

## Supplementary material

Figure S1 **Plasmid maps of chromosomal integrative plasmids used in this study**. Resistance cassettes are shown in red color while the genetic GFP+fusion constructs are shown in green. Homologous flanks allowing integration of the plasmids into the *S. mutans* chromosome are shown in gray color.

Figure S2 **Fluorescence microscopic images of strains MR30-34 growing in complex THBY medium**. Strains MR30-34, carrying *gfp*+ in different genomic loci (agaL, bacA1, SMU_1405, lacE, SMU_1577) and under control of the glycosyltransferase B (gtfB) promoter were grown to an OD_600_ of 0.8. Cells were collected, washed and analyzed using florescence microscopy. Fluorescence images of the strains and a non-fluorescing wild-type strain (control) are presented.

Figure S3 **Visualization of *de novo* cell wall synthesis in Carolacton treated and untreated *S. mutans* wild-type cells**. Untreated and Carolacton treated (5.3 μM) *S. mutans* cells were grown in complex THBY medium to an OD_600_ of 0.5. Subsequently cells were stained with 1 μM Bodipy-Fl vancomycin for 30 min. Cells were harvested, washed and analyzed using fluorescence microscopy. The fluorescence microscopic images of Carolacton treated **(A)** and untreated **(B)** cells are shown.

Figure S4 **Temporal development of fluorescence intensity for two chromosomal reporter strains constitutively expressing GFP+DivIVa**. Reporter strains MR35 and MR49 were grown in complex THBY medium to early exponential growth phase (OD_600_ = 0.2) and GFP+-DivIVa expression was recorded every 30 min for 3 h. Cells were collected, washed and analyzed using flow cytometry. Line plots of the relative fluorescence intensity in course of time of strains MR35 (red), MR49 (blue) and the non-fluorescing *S. mutans* WT cells (black) are shown. For the generation of line plots the GFP fluorescence of 50,000 individual cells was recorded. The mean and the standard deviation of 3 independent biological replicates are presented.

Figure S5 **Dose response curve of the XylS1 promoter cassette to inducer D-xylose in strain MR39**. The Gfp+-DivIVa reporter strain MR39 was grown in complex THBY medium to an OD_600_ of 0.2. Cells were split in several identical aliquots and treated with different concentrations of D-xylose ranging from 0 to 2.66^*^10^5^ μM. 3 h post induction cells were collected, washed, sonicated and analyzed using flow cytometry. The relative GFP fluorescence intensity of 50000 individual cells was measured for each sample. The corresponding dose response curve is shown in part **(A)**. In part **(B)** the dose response is plotted in the logarithmic scale for the inducer concentration (black curve) and the fitted dose response curve assuming sigmoidal dose-response behavior is shown (red line). Based on the fitted curve the EC_50_ value was calculated using the software Origin 9.0.

Figure S6 **Effect of Carolacton treatment on the localization of cell division protein DivIVa in *S. mutans* UA159**. The chromosomal GFP+-DivIVa reporter strain MR40 carrying the xylose inducible XylS2 promoter cassette was grown in buffered (75 mM and pH 6.5) complex THBY medium to the early exponential growth phase (OD_600_ = 0.2). Cells were treated with and without 5.3 μM Carolacton and GFP+-DivIV expression was induced for all samples (treated/untreated) with 1.5% D-xylose. 3 h post induction cells were harvested, washed and analyzed under the fluorescence microscope. Phase contrast (column **A**), fluorescence (column **B**) and overlay images (column **C**) of Carolacton treated (upper panel T) and untreated control cells (lower panel **C**) are presented.

Figure S7 **Effect of Carolacton treatment on the localization of cell division protein PknB in *S. mutans* UA159**. The chromosomal GFP+-PknB reporter strain carrying the xylose inducible XylS2 promoter cassette (MR43) was grown in buffered (75 mM and pH 6.5) complex THBY medium to the early exponential growth phase (OD_600_ = 0.2). Cells were treated with and without 5.3 μM Carolacton and GFP+-PknB expression was induced for all samples (treated/untreated) with 1.5% D-xylose. 3 h post induction cells were harvested, washed and analyzed under the fluorescence microscope. Phase contrast (column **A**), fluorescence (column **B**) and overlay images (column **C**) of Carolacton treated (upper panel T) and untreated control cells (lower panel **C**) are presented.

Figure S8 **THMM prediction of transmembrane helices for the hypothetical proteins SMU_503 and SMU_609**. The amino acid sequence of the proteins was analyzed using the TMHMM server version 2.0. The plot shows the posterior probabilities of inside/outside/transmembrane helix for each residue.

Table S1 **Plasmids used in this study**.
