# Supplementary figures and images for: Carolacton Treatment Causes Delocalization of the Cell Division Proteins PknB and DivIVa in Streptococcus mutans in vivo
Source: Front Microbiol. 2016 May 11;7:684. doi: 10.3389/fmicb.2016.00684 (PMC4862990; doi:10.3389/fmicb.2016.00684)

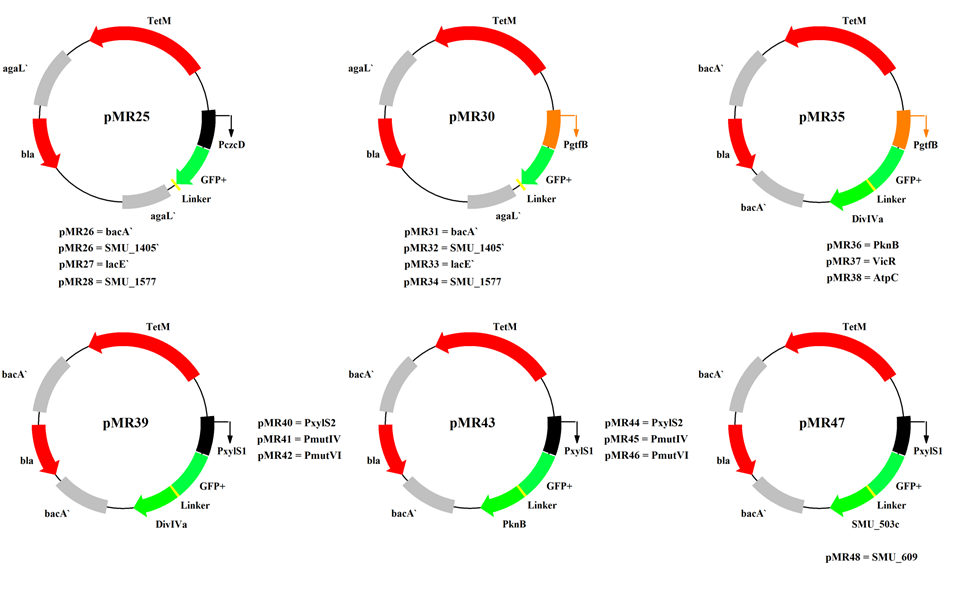

Supplement: Figure S1 — Plasmid maps of chromosomal integrative plasmids used in this study. Resistance cassettes are shown in red color while the genetic GFP+fusion constructs are shown in green. Homologous flanks allowing integration of the plasmids into the S. mutans chromosome are shown in gray color. [file Image1.TIF]

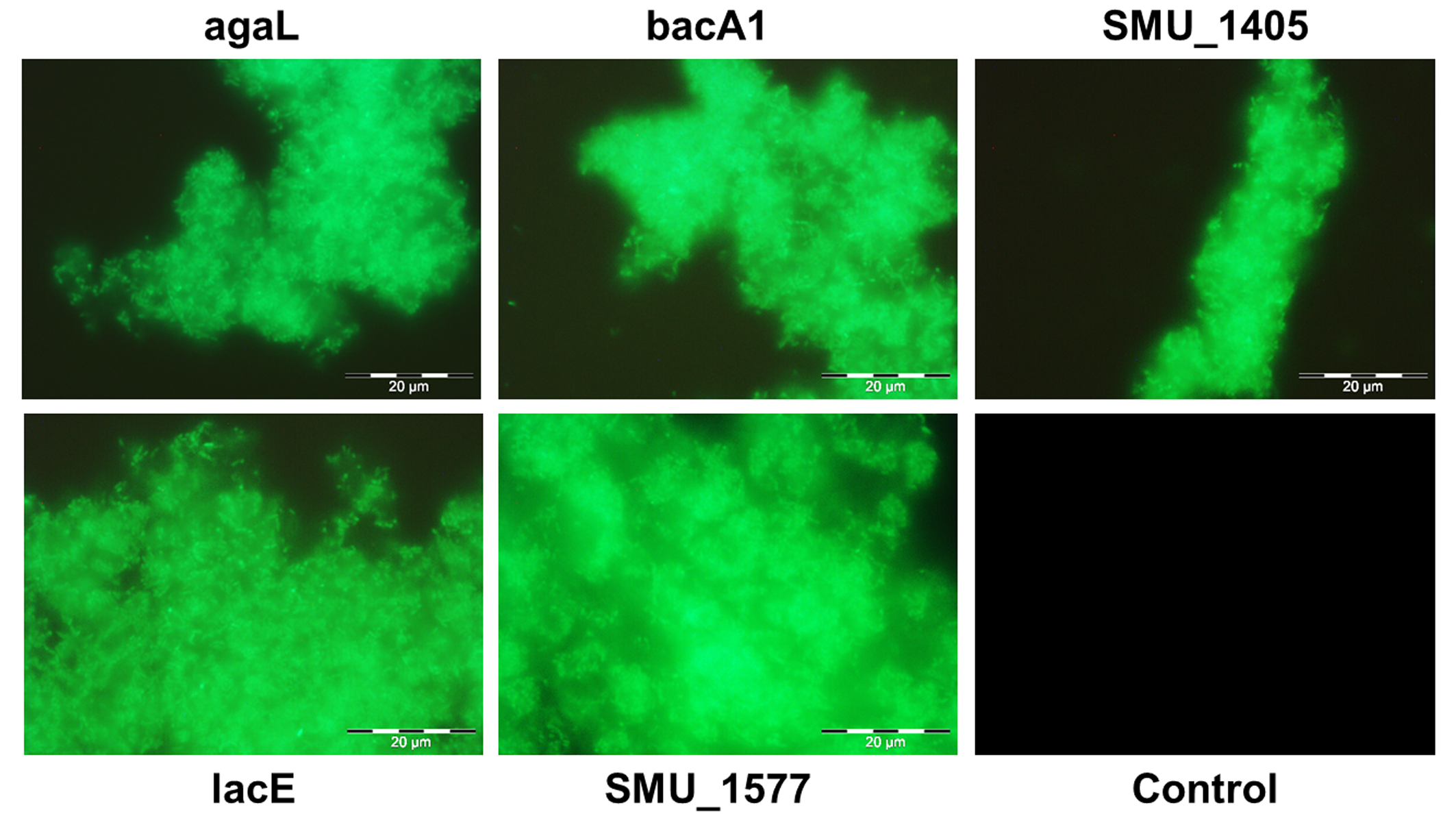

Supplement: Figure S2 — Fluorescence microscopic images of strains MR30-34 growing in complex THBY medium. Strains MR30-34, carrying gfp+ in different genomic loci (agaL, bacA1, SMU_1405, lacE, SMU_1577) and under control of the glycosyltransferase B (gtfB) promoter were grown to an OD600 of 0.8. Cells were collected, washed and analyzed using florescence microscopy. Fluorescence images of the strains and a non-fluorescing wild-type strain (control) are presented. [file Image2.TIF]

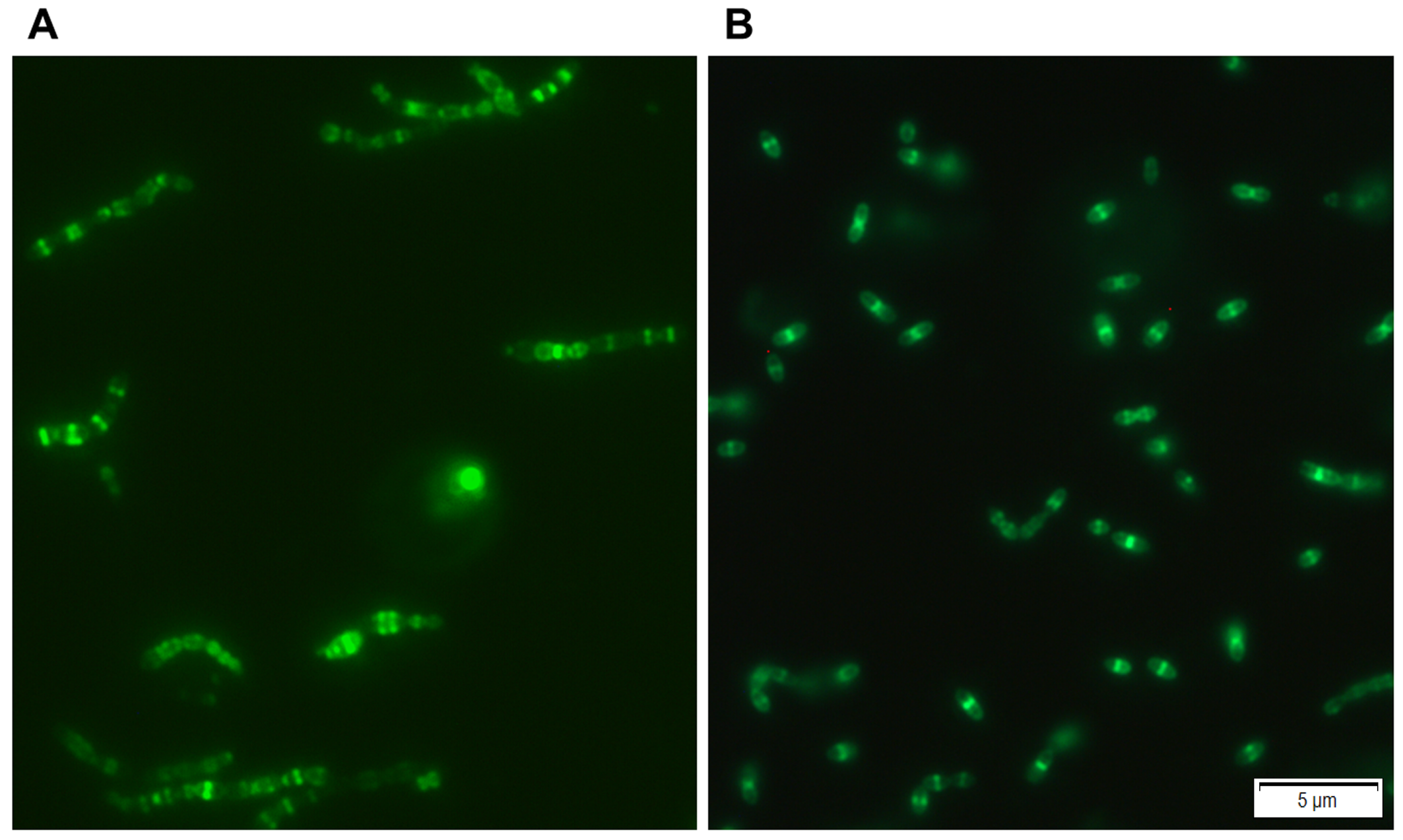

Supplement: Figure S3 — Visualization of de novo cell wall synthesis in Carolacton treated and untreated S. mutans wild-type cells. Untreated and Carolacton treated (5.3 μM) S. mutans cells were grown in complex THBY medium to an OD600 of 0.5. Subsequently cells were stained with 1 μM Bodipy-Fl vancomycin for 30 min. Cells were harvested, washed and analyzed using fluorescence microscopy. The fluorescence microscopic images of Carolacton treated (A) and untreated (B) cells are shown. [file Image3.TIF]

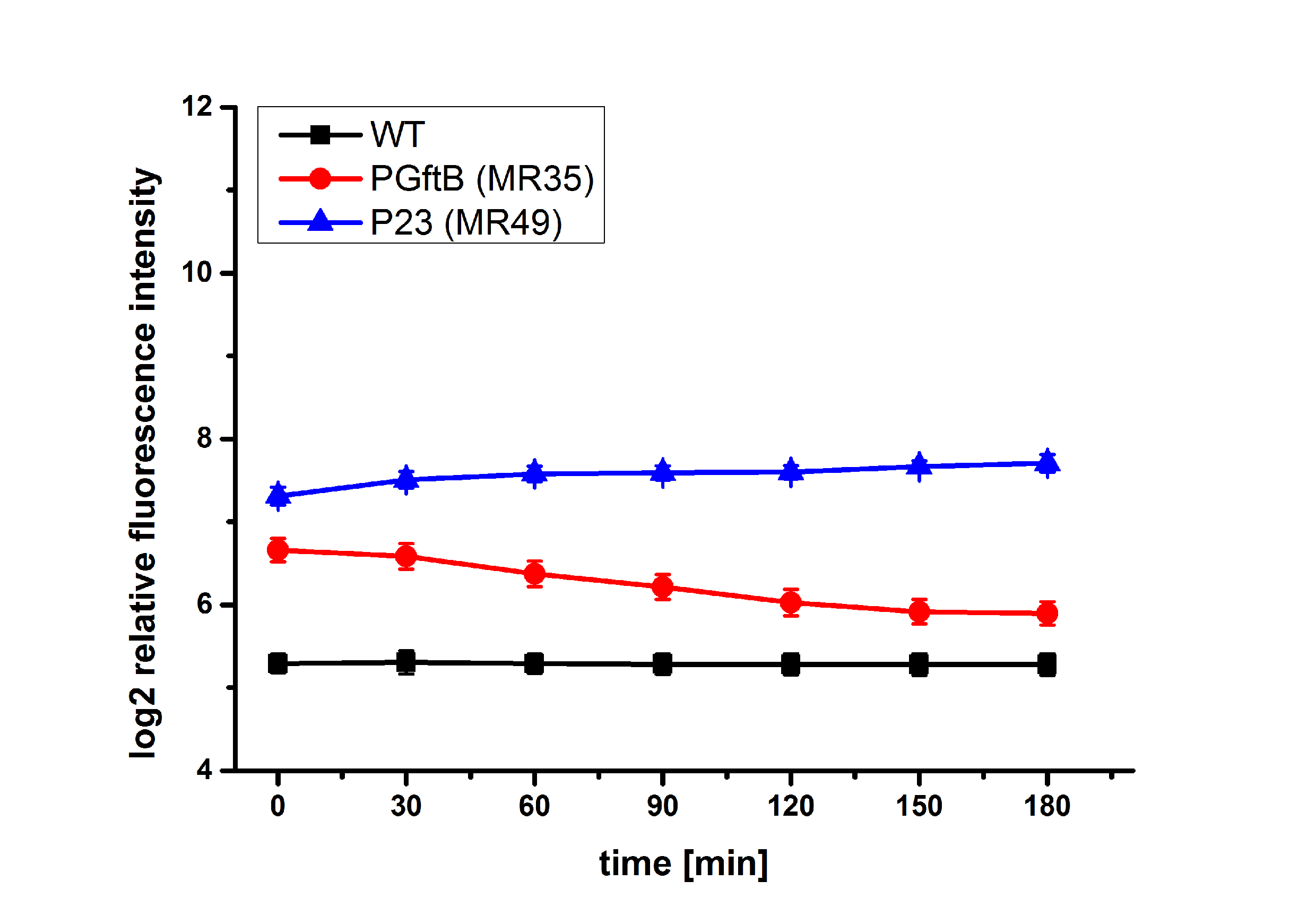

Supplement: Figure S4 — Temporal development of fluorescence intensity for two chromosomal reporter strains constitutively expressing GFP+DivIVa. Reporter strains MR35 and MR49 were grown in complex THBY medium to early exponential growth phase (OD600 = 0.2) and GFP+-DivIVa expression was recorded every 30 min for 3 h. Cells were collected, washed and analyzed using flow cytometry. Line plots of the relative fluorescence intensity in course of time of strains MR35 (red), MR49 (blue) and the non-fluorescing S. mutans WT cells (black) are shown. For the generation of line plots the GFP fluorescence of 50,000 individual cells was recorded. The mean and the standard deviation of 3 independent biological replicates are presented. [file Image4.TIF]

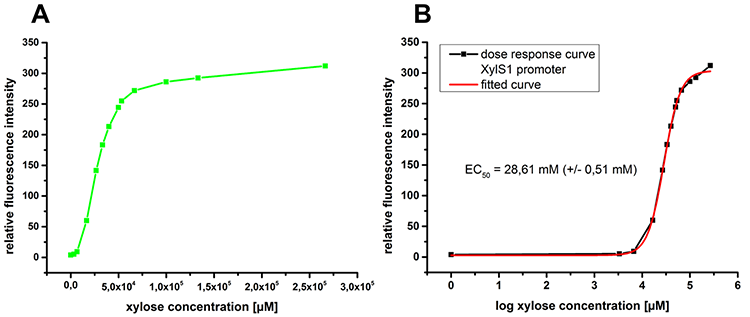

Supplement: Figure S5 — Dose response curve of the XylS1 promoter cassette to inducer D-xylose in strain MR39. The Gfp+-DivIVa reporter strain MR39 was grown in complex THBY medium to an OD600 of 0.2. Cells were split in several identical aliquots and treated with different concentrations of D-xylose ranging from 0 to 2.66*105 μM. 3 h post induction cells were collected, washed, sonicated and analyzed using flow cytometry. The relative GFP fluorescence intensity of 50000 individual cells was measured for each sample. The corresponding dose response curve is shown in part (A). In part (B) the dose response is plotted in the logarithmic scale for the inducer concentration (black curve) and the fitted dose response curve assuming sigmoidal dose-response behavior is shown (red line). Based on the fitted curve the EC50 value was calculated using the software Origin 9.0. [file Image5.tif]

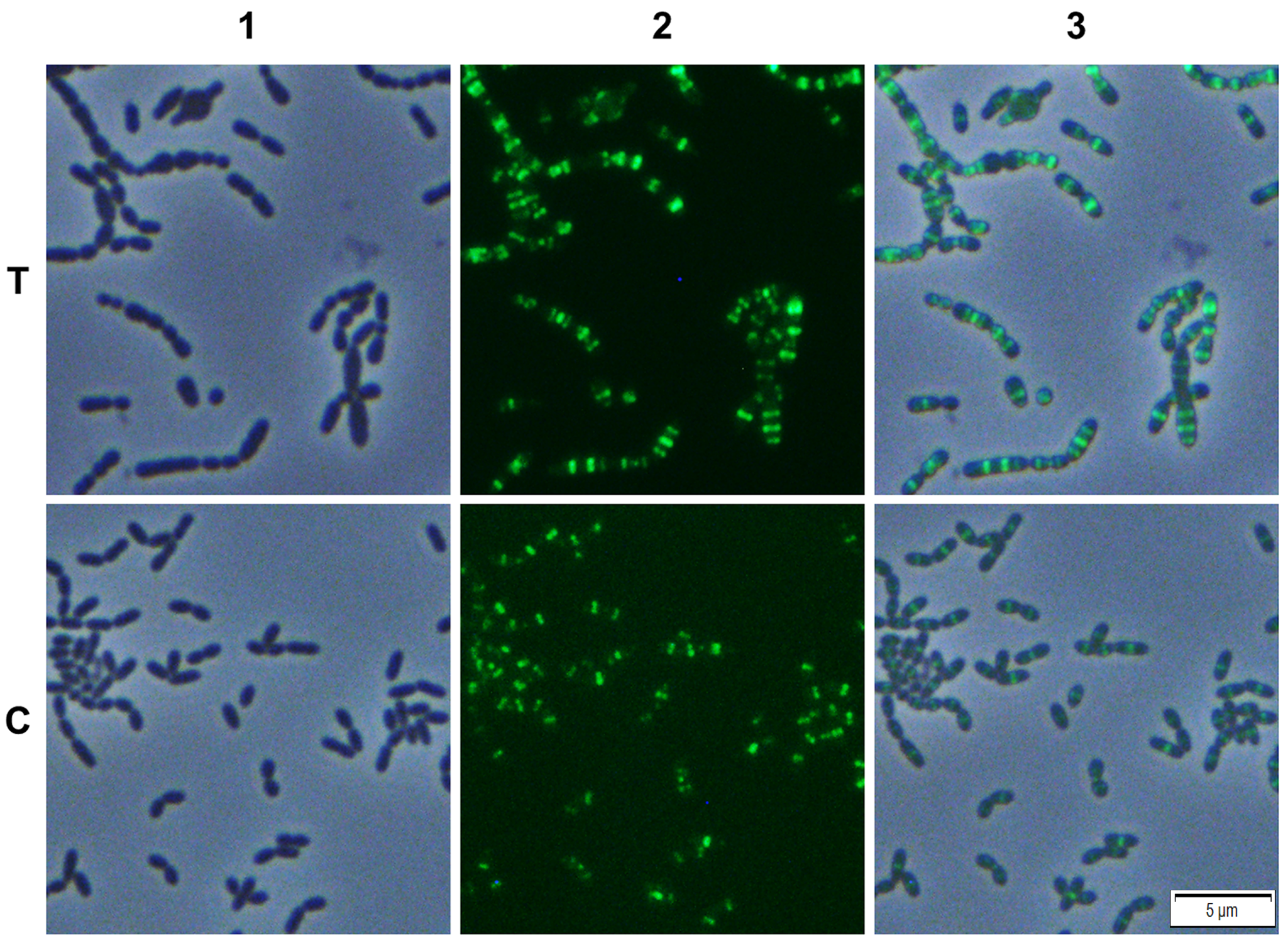

Supplement: Figure S6 — Effect of Carolacton treatment on the localization of cell division protein DivIVa in S. mutans UA159. The chromosomal GFP+-DivIVa reporter strain MR40 carrying the xylose inducible XylS2 promoter cassette was grown in buffered (75 mM and pH 6.5) complex THBY medium to the early exponential growth phase (OD600 = 0.2). Cells were treated with and without 5.3 μM Carolacton and GFP+-DivIV expression was induced for all samples (treated/untreated) with 1.5% D-xylose. 3 h post induction cells were harvested, washed and analyzed under the fluorescence microscope. Phase contrast (column A), fluorescence (column B) and overlay images (column C) of Carolacton treated (upper panel T) and untreated control cells (lower panel C) are presented. [file Image6.TIF]

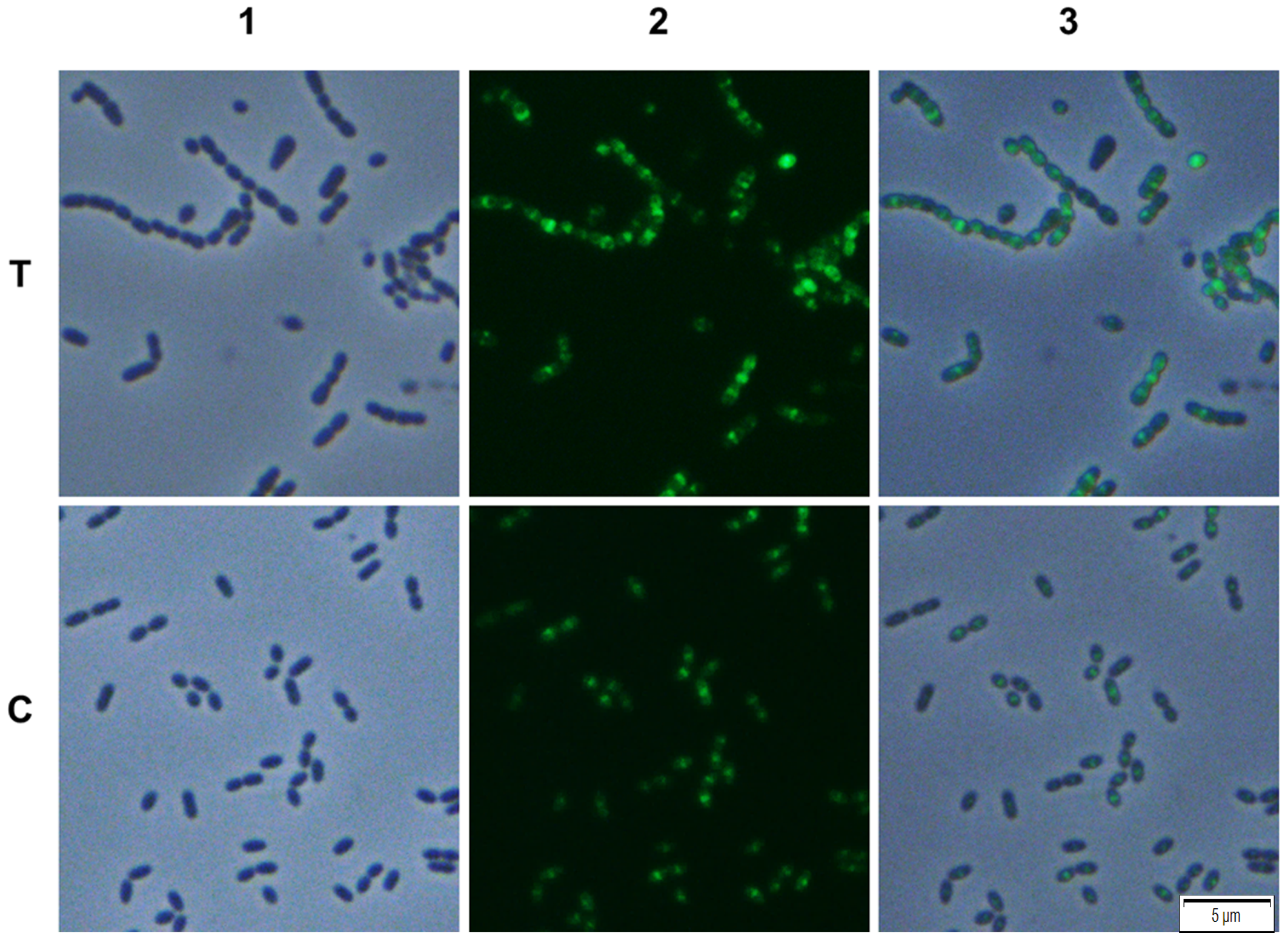

Supplement: Figure S7 — Effect of Carolacton treatment on the localization of cell division protein PknB in S. mutans UA159. The chromosomal GFP+-PknB reporter strain carrying the xylose inducible XylS2 promoter cassette (MR43) was grown in buffered (75 mM and pH 6.5) complex THBY medium to the early exponential growth phase (OD600 = 0.2). Cells were treated with and without 5.3 μM Carolacton and GFP+-PknB expression was induced for all samples (treated/untreated) with 1.5% D-xylose. 3 h post induction cells were harvested, washed and analyzed under the fluorescence microscope. Phase contrast (column A), fluorescence (column B) and overlay images (column C) of Carolacton treated (upper panel T) and untreated control cells (lower panel C) are presented. [file Image7.TIF]

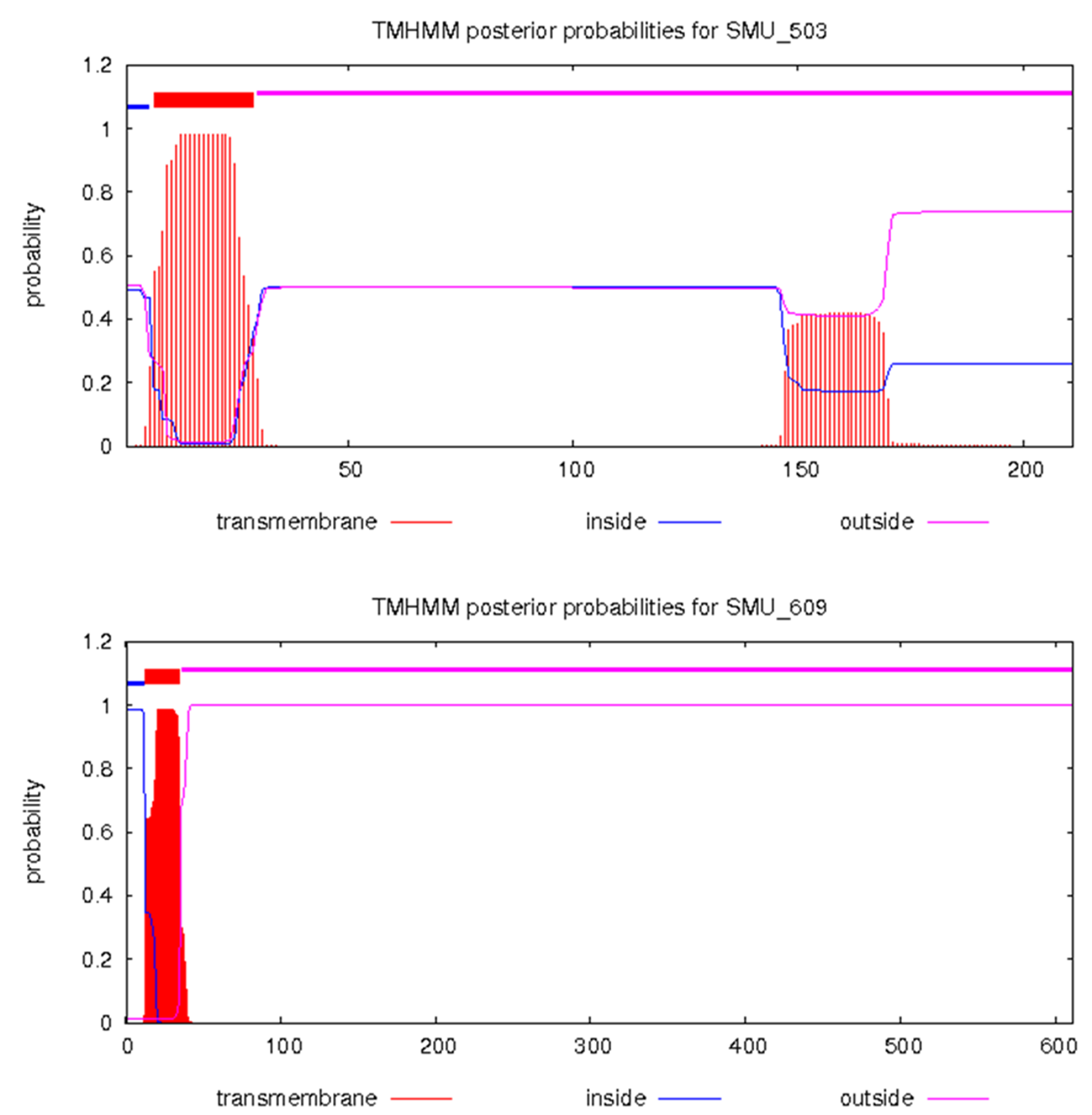

Supplement: Figure S8 — THMM prediction of transmembrane helices for the hypothetical proteins SMU_503 and SMU_609. The amino acid sequence of the proteins was analyzed using the TMHMM server version 2.0. The plot shows the posterior probabilities of inside/outside/transmembrane helix for each residue. [file Image8.TIF]
