# Supplementary material for: Carolacton Treatment Causes Delocalization of the Cell Division Proteins PknB and DivIVa in Streptococcus mutans in vivo
Source: Front Microbiol. 2016 May 11;7:684. doi: 10.3389/fmicb.2016.00684 (PMC4862990; doi:10.3389/fmicb.2016.00684)
Supplement: Table S1 — Plasmids used in this study. [file Table1.docx]

**Table S1: Primers used in this study**

| **name** | **Sequence** | **purpose** |
| --- | --- | --- |
| P1_EX1 | Ccactagttccagctttagctgcagcttct | amplification pJWV25 |
| P2_EX1 | Cacatgcagctcccggagacggtca | amplification pJWV25 |
| D_agaL_For | Agctggaactagtggagcggccgcctgacagaaaatacgattgattatgttaaatg | downstream flank *agaL* |
| D_agaL_Rev | ccgggagctgcatgtgggcgcgccgtattgtttactcaaataatcaccttg | downstream flank *agaL* |
| D_bacA_For | gctggaactagtggagcggccgcgatttatttgaaactgaatcagctaaaag | downstream  flank *bacA1* |
| D_bacA_Rev | ccgggagctgcatgtgggcgcgccacggaaataatttttagtgactaaatc | downstream flank *bacA1* |
| D_1405_For | gctggaactagtggagcggccgcgcttattcctcgaaaaacgaagaaattttat | downstream flank SMU_1405 |
| D_1405_Rev | ccgggagctgcatgtgggcgcgccattgagatcaatccgcgtttcataaag | downstream flank SMU_1405 |
| D_1577_For | gctggaactagtggagcggccgcagtgaattaaataataattattatgaagc | downstream flank SMU_1577 |
| D_1577_Rev | ccgggagctgcatgtgggcgcgcccctcatcatttggatctatatcaaa | downstream fl. SMU_1577 |
| D_lacE_For | gctggaactagtggagcggccgcatccaatacgatcttgccaaggttttcca | downstream flank *lacE* |
| D_lacE_Rev | ccgggagctgcatgtgggcgcgccgataacgctcttgcgtatcaaaatcaaca | downstream flank *lacE* |
| P1_EX2 | Aattaattcttgaagacgaaagggcctcgt | amplification vector pMR20-24 |
| P2_EX2 | Gctagctttacagacaaagaactatcc | amplification vector pMR20-24 |
| U_lacE_For | tcttcaagaattaattggccggccaatatttatctacgtgctattcgtgatg | upstream flank *lacE* |
| U_lacE_Rev | tgtctgtaaagctagcggccggccgaaagttggcacaacagaagcacgtcca | upstream flank *lacE* |
| U_agaL_For | tcttcaagaattaattggccggccttattttggatttcttttctaaatttactgg | upstream flank *agaL* |
| U_agaL_Rev | tgtctgtaaagctagcggccggcctaaagaaaatgaatattctaaatatctat | upstream flank agaL |
| U_bacA1_For | tcttcaagaattaattggccggcctgatgttacttcaatggatatttttgttag | upstream  flank *bacA1* |
| U_bacA1_Rev | tgtctgtaaagctagcggccggcctagtaatattaaaaatcaaattgtgcta | upstream flank *bacA1* |
| U_1405_For | tcttcaagaattaattggccggcctcgtttattgttgcaatttattaaagag | upstream flank SMU_1405 |
| U_1405_Rev | tgtctgtaaagctagcggccggcctactgttcttatcttaataaattatcc | upstream flank SMU_1405 |
| U_1577_For | tcttcaagaattaattggccggccctaattgcacgatgagctgtcccgtatc | upstream flank SMU_1577 |

| U_1577_Rev | tgtctgtaaagctagcggccggccgttggattatgaaaacgtatgagaaaat | upstream flank SMU_1577 |
| --- | --- | --- |
| P1_MR25-29 | Cacagagtcagcaagaacagcgctagaac | vector ampl. pMR25-29 |
| P2_MR25-29 | Tctagaggatctggtggagaagctgcagct | vector ampl.  pMR25-29 |
| PGtfB_For | Cgcaagtaactaactctgtcgtttca | amplification GtfB promoter |
| PGtfB_Rev | agcttgtccattaggaacctccaaattttaaactgtttta | amplification GtfB promoter |
| GFP+_For | ggaggttcctaatggacaagcttagcaaaggagaagaactt | amplification GFP+ |
| GFP+_Rev2 | Ttatgcggccgctccactagttccagct | ampl. GFP+ without stop |
| GFP+_Rev1 | Ttatttgtagagctcatccatgccatgtgta | amplification GFP with stop |
| PgtfB_GFP_For | cttgctgactctgtgggccggcccgcaagtaactaactctgtcgtttcattg | ampl. insert PgtfB-GFP+ |
| 1342-GFPRev | caccagatcctctagattatttgtagagctcatccatgccatgtgtaat |  |
| PgtfB_GFP_Rev | caccagatcctctagatttgtagagctcatccatgccatgtgtaatc | ampl. insert PgtfB-GFP+ |
| P1_WV | Tccagctttagctgcagcttctccaccaga | amplification vector pMR31 |
| P2_WV | ggagcggccgcgatttatttgaaactgaatc | amplification vector pMR31 |
| WVDivIVaFor | tgcagctaaagctggagcaattacagcacttgaaattaaagataaaacatttgg | amplification *divIVa* |
| WVDivIVaRev | aaatcgcggccgctccactagtttattcgttaatatttaatttaaaagtctgtgt | amplification *divIVa* |
| WVPknBFor | tgcagctaaagctggaattcagattggcaaattatttgctggtcgt | amplification *pknB* |
| WVPknBRev | aaatcgcggccgctccactagtttaatgtgaagttgtcgtcgcacttgaactag | amplification *pknB* |
| WVVicRFor | tgcagctaaagctggaaagaaaattctaatcgttgacgatgaaaaacc | amplification *vicR* |
| WVVicRRev | aaatcgcggccgctccactagtttagtcatatgatttcatgtaataaccaac | amplification *vicR* |
| SMU503_For | tgcagctaaagctggaacacctaaaaaaatcaaaatagctctaacagc | amplification SMU_503 |
| SMU503_Rev | aaatcgcggccgctccactagtttactgcttgtttaatgatactggataggga | amplification SMU_503 |
| atpC_For | gcagctaaagctggaacaggctctcttagtgaaatcaaggtaag | amplification *atpC* |
| atpC_Rev | aatcgcggccgctccactagtttagtctagggcattggcacccgca | amplification *atpC* |
| SMU_609_For | gcagctaaagctggaaaaagaattgatattaatcatcaagcacaac | amplification SMU_609 |
| SMU_609_Rev | aatcgcggccgctccactagttcaatcaatgataatataacgacgaat | amplification SMU_609 |

| P1_PROMO | Cacagagtcagcaagaacagcgctagaacc | promoter exch. in pMR31 |
| --- | --- | --- |
| P2_PROMO | Cttagcaaaggagaagaacttttcactggagttg | promoter exch. in pMR31 |
| PXylS1_For | cttgctgactctgtgcacgaacgaaaatcgatcttctctaacttataggggtaacact | promoter PXylS1 |
| PXylS1_Rev | ttctcctttgctaagtgtattcatatttacctcctttgatttaagtgaacaagt | promoter PXylS1 |
| PXylS2_For | cttgctgactctgtgcacgaacgaaaatcgatcttctctaacttataggggtaacact | promoter PXylS2 |
| PXylS2_Rev | ttctcctttgctaagtgtattcatatttacctcctttgatttaagtgaacaagt | promoter PXylS2 |
| PmutIV_For | cttgctgactctgtgtttgataagcatgcgaacttaaaatacaaatatgg | promoter mutacin IV |
| PmutIV_Rev | ttctcctttgctaagtgtatccatatgataaacacccctttttcatt | promoter mutacin IV |
| PmutVI_For | cttgctgactctgtgtataaaattcccgatttaacttttatcaaag | promoter mutacin VI |
| PmutVI_For | ttctcctttgctaagtgtattcatatgatagatacctcttttccatt | promoter mutacin VI |

**Table S2: Plasmids used in this study**

| Plasmid | Relevant genotype | Reference |
| --- | --- | --- |
| pZX9 | P_gyrA_::xylR::P_ldh_::O_XylA_::luc, Spec | Xie et al. |
| pZX10 | P_gyrA_::xylR::P_XylA_::O_XylA_::luc, Spec | Xie et al. |
| pJWV25 | Amp, tet, bgaA, P_czcD_-gfp+ | Eberhardt et al. |
| pMR25 | Amp, tet, agaL, P_czcD_-gfp+ | This study |
| pMR26 | Amp, tet, bacA1, P_czcD_-gfp+ | This study |
| pMR27 | Amp, tet, SMU_1405, P_czcD_-gfp+ | This study |
| pMR28 | Amp, tet, lacE, P_czcD_-gfp+ | This study |
| pMR29 | Amp, tet, SMU_1577, P_czcD_-gfp+ | This study |
| pMR30 | Amp, tet, agaL, P_gtfB_-gfp+ | This study |
| pMR31 | Amp, tet, bacA1, P_gtfB_-gfp+ | This study |
| pMR32 | Amp, tet, SMU_1405, P_gtfB_-gfp+ | This study |
| pMR33 | Amp, tet, lacE, P_gtfB_-gfp+ | This study |
| pMR34 | Amp, tet, SMU_1577, P_gtfB_-gfp+ | This study |
| pMR35 | Amp, tet, bacA1, P_gtfB_-gfp+-divIVa | This study |
| pMR36 | Amp, tet, bacA1, P_gtfB_-gfp+-pknB | This study |
| pMR37 | Amp, tet, bacA1, P_gtfB_-gfp+-vicR | This study |
| pMR38 | Amp, tet, bacA1, P_gtfB_-gfp+-atpC | This study |
| pMR39 | Amp, tet, bacA1, P_XylS1_-gfp+-divIVa | This study |
| pMR40 | Amp, tet, bacA1, P_XylS2_-gfp+-divIVa | This study |
| pMR41 | Amp, tet, bacA1, P_mutIV_-gfp+-divIVa | This study |
| pMR42 | Amp, tet, bacA1, P_mutVI_-gfp+-divIVa | This study |
| pMR43 | Amp, tet, bacA1, P_XylS1_-gfp+-pknB | This study |
| pMR44 | Amp, tet, bacA1, P_XylS2_-gfp+-pknB | This study |
| pMR45 | Amp, tet, bacA1, P_mutIV_-gfp+-pknB | This study |
| pMR46 | Amp, tet, bacA1, P_mutVI_-gfp+-pknB | This study |
| pMR47 | Amp, tet, bacA1, P_XylS1_-gfp+-smu_503 | This study |
| pMR48 | Amp, tet, bacA1, P_XylS1_-gfp+-smu_609 | This study |
